# Nocarimidazoles C and D, antimicrobial alkanoylimidazoles from a coral-derived actinomycete *Kocuria* sp.: application of ^1^*J*_C,H_ coupling constants for the unequivocal determination of substituted imidazoles and stereochemical diversity of anteisoalkyl chains in microbial metabolites

**DOI:** 10.3762/bjoc.16.222

**Published:** 2020-11-05

**Authors:** Md Rokon Ul Karim, Enjuro Harunari, Amit Raj Sharma, Naoya Oku, Kazuaki Akasaka, Daisuke Urabe, Mada Triandala Sibero, Yasuhiro Igarashi

**Affiliations:** 1 Biotechnology Research Center and Department of Biotechnology, Toyama Prefectural University, 5180 Kurokawa, Imizu, Toyama 939-0398, Japan; 2 Shokei Gakuin University, 4-10-1 Yurigaoka, Natori, Miyagi 981-1295, Japan; 3 Department of Marine Science, Faculty of Fisheries and Marine Science, Diponegoro University, Semarang, Central Java 50275, Indonesia

**Keywords:** ^1^*J*_C,H_, alkanoylimidazoles, anteiso, *Kocuria*, nocarimidazole

## Abstract

Chemical investigation of secondary metabolites from a marine-derived actinomycete strain of the genus *Kocuria*, isolated from a stony coral *Mycedium* sp., led to the identification of two new alkanoylimidazoles, nocarimidazoles C (**1**) and D (**2**) as well as three known congeners, nocarimidazoles A (**3**) and B (**4**) and bulbimidazole A (**5**). Structure analysis of **1** and **2** by NMR and MS revealed that both are 4-alkanoyl-5-aminoimidazoles with a 6-methyloctanoyl or decanoyl chain, respectively. Two possible positions of the amino group on the imidazole rings (C-2 and C-5) posed a challenge in the structure study, which was settled by the measurement of ^1^*J*_C,H_ coupling constants for comparison with those of synthetically prepared model imidazoles. The absolute configurations of the anteisoalkanoyl group present in **1**, **4**, and **5** were determined by low-temperature HPLC analysis of the degradation products labeled with a chiral anthracene reagent, which revealed that **1** is a mixture of the *R*- and *S*-enantiomers with a ratio of 73:27, **4** is the pure (*S*)-enantiomer, and **5** is the (*S*)-enantiomer with 98% ee. The present study illustrates the diversity in the stereochemistry of anteiso branching in bacterial metabolites. Compounds **1**−**4** were moderately antimicrobial against Gram-positive bacteria and fungi, with MIC ranges of 6.25–25 μg/mL.

## Introduction

The phylum *Actinobacteria* contains bacterial genera most prolific as producers of novel natural products with high structural diversity, unique modes of action, and potent bioactivities [1]. More than 10,000 secondary metabolites have been isolated from actinomycetes, accounting for almost 45% of all known microbial secondary metabolites. Particularly, 70% of them were isolated from the genus *Streptomyces*, the dominant genus commonly found in terrestrial environments [2]. The number of new bioactive compounds from actinomycetes, especially those from terrestrial sources, is likely reaching a plateau after intensive screening activities over several decades [3]. Actinomycetes also inhabit marine environments, including seashores, coastal waters, and bottom sediments or can be found in association with marine organisms such as invertebrates and plants [4–5]. Marine actinomycetes show unique physiological adaptations distinct from their terrestrial counterparts in terms of pressure, salinity, or low-temperature tolerance, which might affect their metabolic ability in their habitat [6–7]. Their secondary metabolite machinery is activated in the sea, as indicated by the isolation of enediyne antitumor antibiotics from marine invertebrates. Namenamicin [8] and the shishijimicins [9], the chalicheamicin-type enediyne polyketides, were isolated from a colonial tunicate. These compounds are considered to be the metabolites of bacterial endosymbionts that internalized biosynthetic genes, likely to have evolved in an actinomycete or an ancestral bacterium in the same lineage. Further exploration of marine habitats has been disclosing a number of unprecedented molecules of actinomycete origin. For example, salinosporamide A, an antitumor drug candidate in clinical trials, is a proteasome inhibitor with an unusual γ-lactam-β-lactone bicyclic core produced by marine *Salinospora tropica* [10]. Abyssomicin, another example of uncommon polycyclic frameworks, is an antibacterial metabolite of marine *Verrucosispora*, effective against methicillin-resistant *Staphylococcus aureus* (MRSA) and *Mycobacterium tuberculosis* [11–12]*.*

The genus *Kocuria*, formerly categorized in the genus *Micrococcus*, is a Gram-positive unicellular coccus belonging to the family *Micrococcaceae* [13]. Members of the genus *Kocuria* have been isolated from diverse marine environments such as seawater [14–15], sediments [16], and deep-sea hydrothermal plumes [17]. The genome size of the genus *Kocuria* is around 2.7 to 3.0 Mbp in average; which is one of the smallest among actinomycetes. However, the latest genomic information suggests the presence of biosynthetic genes for nonribosomal peptide synthetase and type III polyketide synthase in some *Kocuria* strains [18], which leaves a hope for new secondary metabolites. At present, only a few limited structural types of metabolites, including polyamine-derived siderophores and modified peptides, are known from *Kocuria* and *Micrococcus* [19–20].

In our continuing investigation on secondary metabolites from marine bacteria, five alkanoylimidazoles were obtained from the culture extract of a *Kocuria* strain isolated from a stony coral. Alkanoylimidazoles are a new and rare class of natural products, first described in 2015 by Fenical et al. from marine *Nocardiopsis* [21] and were recently found from a marine obligate bacterium *Microbulbifer* by our group [22]. We herein report the isolation, structure determination, and biological activities of two new alkanoylimidazoles, nocarimidazoles C (**1**) and D (**2**), along with the identification of three known related compounds, nocarimidazoles A (**3**) and B (**4**) as well as bulbimidazole A (**5**). We also discuss the stereochemical diversity of the anteisoalkanoyl group in these compounds.

## Results and Discussion

**Figure 1:**
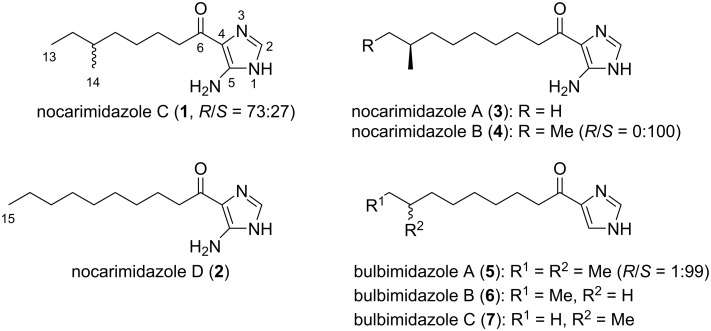
Structure of the nocarimidazoles **1**–**4** and the bulbimidazoles **5**–**7**.

Strain T35-5 was isolated from the scleractinian coral of the genus *Mycedium*, collected near the coast of Karimunjawa, Central Java, Indonesia. Analysis of the 16S rRNA gene sequence identified the producing strain as a member of the genus *Kocuria*. The whole culture broth of *Kocuria* sp. T35-5, cultured in A11M seawater medium at 30 °C for five days, was extracted with 1-butanol, and the extract was subjected to chromatographic purification, yielding two new alkanoylimidazoles, nocarimidazole C (**1**) and D (**2**), along with three known congeners, nocarimidazole A (**3**) and B (**4**) as well as bulbimidazole A (**5**, Figure 1).

Nocarimidazole C (**1**) was obtained as a pale yellow amorphous solid. The molecular formula was determined as C_12_H_21_N_3_O based on a protonated molecular ion [M + H]^+^ at *m*/*z* 224.1763 (Δ = +0.6 mmu). The four degrees of unsaturation, calculated from the molecular formula, and the UV absorption band around 296 nm indicated the presence of a conjugated system in this molecule. The IR absorption bands at 3127 and 1664 cm^−1^ implied the presence of OH/NH and carbonyl groups, respectively. The ^1^H NMR spectrum was rather simple, displaying only 6 signals: a deshielded proton singlet resonance, three isolated aliphatic methylene unit resonances, a methylene envelope signal, and a doublet and a triplet methyl group resonance overlapping. The ^13^C NMR spectrum only exhibited several sp^3^ carbon signals at 10–40 ppm, lacking those of sp^2^ carbon atoms in CDCl_3_, CD_3_OD, or DMSO-*d*_6_ (data not shown). The same phenomenon was observed during the study of bulbimidazole A (**5**), which did not show sp^2^ carbon signals in neutral solutions due to the presence of multiple resonance structures for the imidazole moiety [22]. We envisaged that due to the presence of an imidazole ring, the UV spectra of **1** and **5** would obviously be different, and as expected, supplementation of a trace amount of trifluoroacetic acid (TFA) to DMSO-*d*_6_, combined with a longer relaxation delay (*d1* = 30 s) for the ^13^C NMR experiment, greatly improved the detectability of sp^2^ carbon resonances. The ^13^C and HSQC spectra collected in this solvent mixture allowed the assignment of 12 carbon signals to one deshielded carbonyl carbon atom (δ_C_ 189.4), two nonprotonated sp^2^ carbon atoms (δ_C_ 109.7, 144.6), one sp^2^ methine unit (δ_C_ 130.9), one sp^3^ methine carbon atom, five sp^3^ methylene units, and two methyl moieties (Table 1).

**Table 1:** ^1^H and ^13^C NMR spectroscopic data for nocarimidazoles C (**1**) and D (**2**) in DMSO-*d*_6_ with TFA.

	**1**				**2**		
			
no.	δ_C_^a^	δ_H_ mult (*J* in Hz)^b^	HMBC^b,c^		δ_C_^a^	δ_H_ mult (*J* in Hz)^b^	HMBC^b,c^

2	130.9, CH	8.62, s	4, 5, 6		130.9, CH	8.59, s	4, 5, 6
4	109.7, C				109.7, C		
5	144.6, C				144.7, C		
6	189.4, C				189.3, C		
7	38.3, CH_2_	2.66, t (7.4)	6, 8, 9		38.3, CH_2_	2.66, t (7.4)	4, 6, 8, 9
8	23.9, CH_2_	1.55, quint (7.2)	6, 10		23.6, CH_2_	1.54, quint (7.3)	6, 7, 9, 10
9	26.2, CH_2_	1.27^d^	11		28.76, CH_2_	1.22–1.26^d^	7
10	35.9, CH_2_	1.07, m	9, 11		29.1, CH_2_	1.22–1.25^d^	8
		1.27^d^	9, 11				
11	33.8, CH	1.27^d^	9, 13, 14		29.0,^e^ CH_2_	1.22–1.25^d^	9, 10, 12
12	29.0, CH_2_	1.08, m	11, 13, 14		28.83,^e^ CH_2_	1.22–1.26^d^	
		1.26^d^	11, 13, 14				
13	11.3, CH_3_	0.80, t (7.4)	11, 12		31.4, CH_2_	1.21,^d^ m	14
14	19.2, CH_3_	0.79, d (6.7)	10, 11, 12		22.3, CH_2_	1.21–1.25^d^	13, 15
15					14.0, CH_3_	0.83, t (6.8)	13, 14

^a^Recorded at 125 MHz (reference δ_C_ 39.5). ^b^Recorded at 500 MHz (reference δ_H_ 2.49). ^c^From the proton stated to the indicated carbon atom(s). ^d^Overlapping signals. ^e^Assignment may be interchangeable.

**Figure 2:**
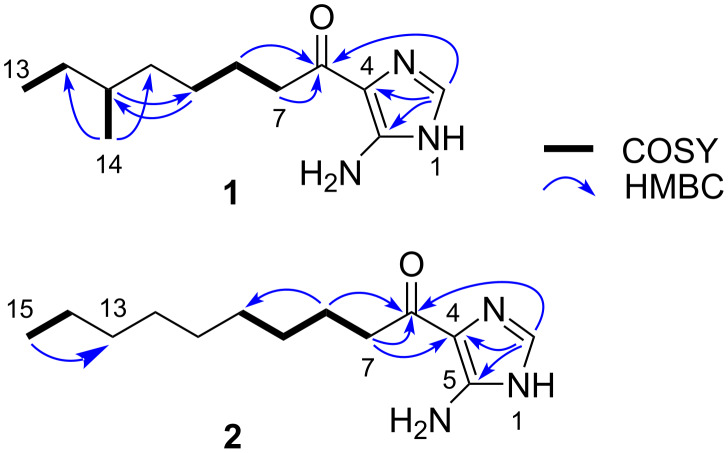
COSY and key HMBC correlations for **1** and **2**.

Three small fragments (H-7/H-8/H-9, H-11/H-14, H-12/H-13) were defined by the analysis of the COSY spectrum (Figure 2). Meanwhile, HMBC correlations from the two methyl protons (H-13 and H-14) to well-resolved C-11, and C-12, along with a correlation from H-14 to C-10, allowed to assemble an anteisomethyl terminus from C-10 to C-14. The connectivity between C-9 and C-10 was established by HMBC correlations from H-8 to C-10 and H-10 to C-9. Furthermore, HMBC correlations from H-7 and H-8 to a deshielded carbonyl carbon atom at δ_C_ 189.4 (C-6) supported a 6-methyloctanoyl substructure. The remaining structural unit has the composition formula C_3_H_4_N_3_ with three double bond equivalents, composed in part by the two nonprotonated sp^2^ carbon atoms (C-4 and C-5) and an sp^2^ methine unit (CH-2) and exhibited HMBC correlations from H-2 to C-4 and C-5. These requirements were only satisfied by an amino-substituted imidazole ring. Indeed, a four-bond correlation from H-2 to C-6 established the linkage between the chain part and the imidazole ring (Figure 2).

**Scheme 1:**
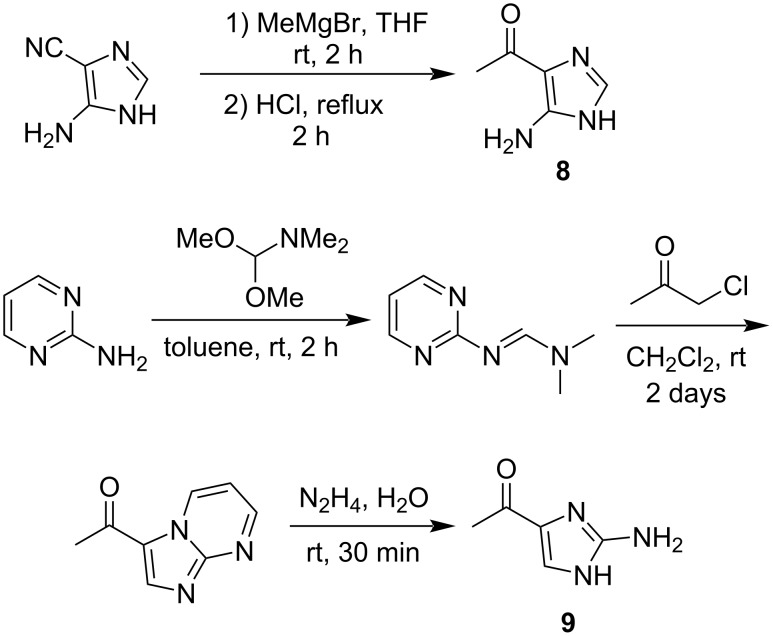
Synthesis of the model compounds **8** and **9**.

**Figure 3:**
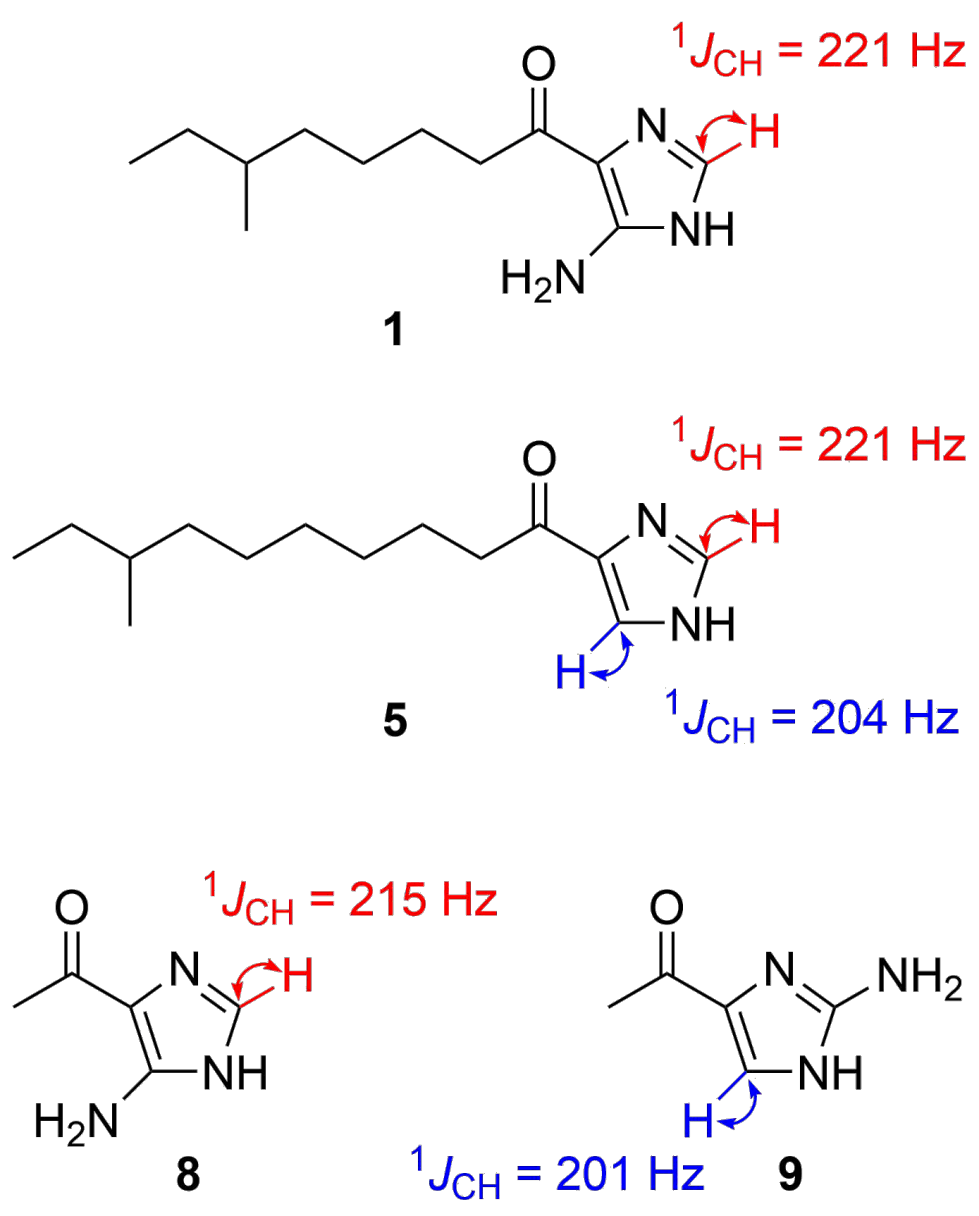
^1^*J*_C,H_ coupling constant for the imidazole ring of the natural products **1** and **5** and the model compounds **8** and **9**.

The remaining question was whether the amino group is bound to C-2 or C-5 in the imidazole ring. A literature survey suggested a diagnostic use of ^1^*J*_C,H_ coupling constants [23]. In imidazole and ʟ-histidine, the ^1^*J*_C,H_ values for H-2/C-2 (208 to ≈222 Hz) were found to be always larger by at least 15 Hz than those for H-4/C-4 (188 to ≈208 Hz) at any pH condition below 11 [24]. Because the sp^2^ methine group in **1** exhibited ^1^*J*_C,H_ = 221 Hz in a coupled HSQC experiment, this was assignable to the imidazole 2-position based on this criterion, and hence a C-5 amino substitution. Additionally, ^1^*J*_C,H_ measurements of bulbimidazole A (**5**) gave 221 Hz for H-2/C-2 and 204 Hz for H-4/C-4 (H-5/C-5 in the numbering system for **5**), which corroborated the assignment. To finally settle this issue, two aminoimidazoles **8** and **9**, possessing 4-acetyl and 5- or 2-amino substitutions, respectively, were synthesized for comparison, according to the reported procedures (Scheme 1) [25–27]. Compound **8** is known as a photolysis product of 6-methylpurine 1-oxide [28], but we prepared it by the Grignard reaction of a commercially available imidazole derivative, 4-isocyano-1*H*-imidazol-5-amine, with MeMgBr. On the other hand, **9** was synthesized in three steps from pyrimidin-2-amine. Imidation of the starting material with 1,1-dimethoxy-*N*,*N*-dimethylmethylamine gave an *N*,*N*- dimethylformimidamide derivative, which was cyclized with 1-chloropropan-2-one to give 3-acetylimidazo[1,2-*a*]pyrimidine, which, following degradation of the pyridimine ring with hydrazine, yielded **9**. The coupled HSQC experiments measured ^1^*J*_C,H_ = 215 Hz for the H-2/C-2 pair in **8** (Figure S26, Supporting Information File 1) and 201 Hz for H-5/C-5 in **9** (Figure S31, Supporting Information File 1). Thus, the amino substitution at C-5 in **1** was unequivocally established (Figure 3).

**Figure 4:**
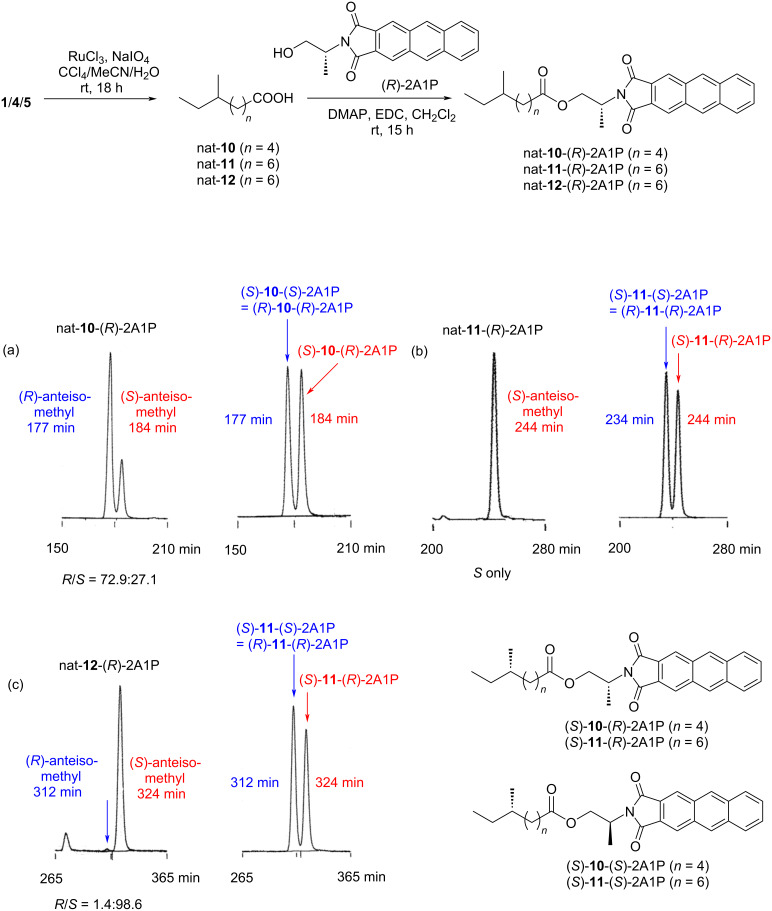
Determination of the absolute configuration of **1** (a), **4** (b), and **5** (c) by the Ohrui–Akasaka method.

The absolute configuration at C-11 in the anteisoalkanoyl chain was determined by the Ohrui–Akasaka method [29]. The imidazole ring was degraded by oxidation using ruthenium tetraoxide and sodium periodate in a biphasic solvent mixture (CCl_4_/MeCN/H_2_O), which gave 6-methyloctanoic acid (*nat*-**10**). The authentic (*S*)-6-methyloctanoic acid and (*S*)-8-methyldecanoic acid were synthesized in our previous studies [22,30]. These anteiso fatty acids were derivatized with a chiral anthracene reagent to yield the esters of (*R*)- or (*S*)-2-(anthracene-2,3-dicarboximido)propanol (*nat*-**10**-(*R*)-2A1P, (*S*)-**10**-(*R*)-2A1P, and (*S*)-**10**-(*S*)-2A1P), which were subjected to HPLC analysis for comparison (Figure 4). The retention times of the standard samples were 177 min for (*S*)-**10**-(*S*)-2A1P (chromatographically equivalent to (*R*)-**10**-(*R*)-2A1P) and 184 min for (*S*)-**10**-(*R*)-2A1P, and *nat*-**10**-(*R*)-2A1P gave both peaks with the area ratio of 72.9:27.1 (Figure 4a). Therefore, **1** was confirmed as an enantiomeric mixture comprising 73% of the *R*- and 27% of the *S*-enantiomer. We also analyzed the absolute configuration of nocarimidazole B (**4**) produced by strain TK35-5. The same compound was originally isolated from marine *Nocadiopsis*, but the absolute configuration was not elucidated. The conversion of **4** into the derivative *nat*-**11**-(*R*)-2A1P, followed by HPLC analysis, revealed that **4** is an enantiomerically pure *S*-enantiomer (Figure 4b). Additionally, the same chiral analysis with bulbimidazole A (**5**) obtained in this study proved the enantiomeric ratio as *R*/*S* = 1.4:98.6 (Figure 4c).

Nocarimidazole D (**2**) was isolated as a pale yellow amorphous solid. The molecular formula of **2** was deduced as C_13_H_23_N_3_O, 14 amu (corresponding to CH_2_) larger than **1**, based on the HRESITOFMS data ([M + H]^+^ at *m*/*z* 238.1915, Δ = +0.2 mmu). The UV and IR spectra showed almost the same features as those for **1**. The interpretation of the ^1^H, ^13^C, and HSQC spectra of **2** in comparison to **1** revealed three additional methylene groups and the absence of one doublet methyl and one methine signal. Two aliphatic fragments, H-7/H-8/H-9 and H-14/H-15, were identified from COSY correlation data. These fragments were then joined into a nonbranching linear alkyl chain by HMBC correlations from H-15 to C-13 and H-8 to C-10, though H-11/C-11 and H-12/C-12 were not completely assignable due to a severe signal overlapping. This alkyl chain was further linked to the imidazole ring through a carbonyl carbon atom C-6 on the basis of HMBC correlations from H-7 and H-2 to C-6 (Figure 2). ^1^H and ^13^C NMR chemical shifts for the imidazole part of **2** were almost the same as those for **1** (Table 1).

The antimicrobial activity of **1**–**4** was tested against Gram-positive bacteria *Kocuria rhizophila* and *Staphylococcus aureus*, Gram-negative bacteria *Escherichia coli* and *Rhizobium radiobacter*, a yeast *Candida albicans*, and two fungi *Glomerella cingulata* and *Trichophyton rubrum* (Table 2). All compounds exhibited moderate activity against Gram-positive bacteria with MICs of 6.25–12.5 μg/mL but were inactive against Gram-negative bacteria. The compounds **1**–**4** were also active against the yeast and fungi, with MIC values ranging from 6.25–25 μg/mL. In addition, the compounds **1** and **4** exhibited weak cytotoxicity against P388 murine leukemia cells, with an IC_50_ of 38 and 33 μM, respectively.

**Table 2:** Antimicrobial activity of the nocarimidazoles **1**–**4**.

	MIC (μg/mL)			
microoroganisms	**1**	**2**	**3**	**4**

*Kocuria rhizophila* ATCC9341	6.25	12.5	6.25	6.25
*Staphylococcus aureus* FDA209P JC-1	12.5	25	12.5	25
*Escherichia coli* NIHJ JC-2	>100	>100	>100	>100
*Rhizobium radiobacter* NBRC14554	>100	>100	>100	>100
*Candida albicans* NBRC0197	25	25	12.5	12.5
*Glomerella cingulata* NBRC5907	12.5	12.5	25	25
*Trichophyton rubrum* NBRC5467	6.25	6.25	25	6.25

## Conclusion

Alkanoylimidazoles, 4-acylated imidazoles of varying chain length and terminal branching, with occasional substitution at C-5 by an amino group, are an emerging class of marine-derived natural products: nocarimidazoles A (**3**) and B (**4**), the first two members in this class, were discovered from a marine actinomycete *Nocardiopsis* [21]; bulbimidazoles A–C (**5**–**7**), on the other hand, were isolated from a marine gammaproteobacterium *Microbulbifer* [22]. In this study, additional members, nocarimidazoles C (**1**) and D (**2**), were obtained from a marine-derived actinomycete of the genus *Kocuria*. The exclusive origin of these metabolites from marine bacteria, as well as the distribution among phylogenetically distinct taxa imply their potential function in the adaptation of the producing organisms to the marine ecosystem.

**Figure 5:**
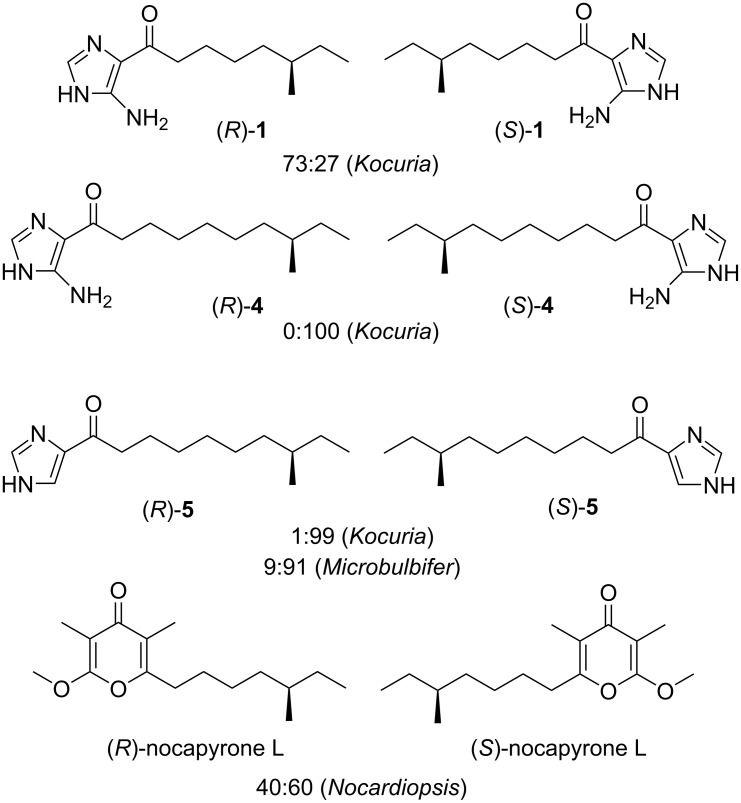
Stereochemical diversity of the anteiso-chain chirality in microbial metabolites.

Similarly to nocarimidazole B (**4**) and bulbimidazole A (**5**), **1** possesses the anteiso-branched alkanoyl chain. The absolute configurations of the anteiso-branched metabolites are in general expected to be *S* in an association to bacterial anteiso fatty acids, which are biosynthesized from ʟ-isoleucine [31–32]. However, we have previously shown that both the anteiso-branched secondary metabolites of marine bacteria, nocapyrone L [30] and bulbimidazole A (**5**) [22], are 2:3 and 9:91 mixtures of the *R*- and *S*-enantiomers, respectively. Again, we encountered enantiomerically mixed anteiso chains in **1** and **5** but at the same time found purely *S*-configured **4** (Figure 5), demonstrating the varied enantiomerical purity and chirality of anteiso-branched bacterial metabolites. Intriguingly, the same chemistry is seen among related compounds from a single organism (e.g., **1**, **4**, and **5**) and even with a compound (in terms of having the common planar structure) from different organisms (e.g., **5**). This lesson not only warrants the insufficiency of assigning the stereochemistry of the synthesized anteiso-chained natural products only by comparison of the optical rotation values but provides a new insight into the structural/biosynthetic diversity in microbial secondary metabolites. Finally, it should be noted that the *R*-enantiomer-rich anteisoalkyl group is extremely rare: only two ceramides, each from the dinoflagellate *Coolia monotis* [33] and the sponge *Ephydatia syriaca* [34], preceed **1**.

## Experimental

### General experimental procedures

The specific rotations were measured on a JASCO P-1030 polarimeter. UV and IR spectra were recorded on a Shimadzu UV-1800 spectrophotometer and a PerkinElmer Spectrum 100 spectrophotometer, respectively. NMR spectra were obtained on a Bruker AVANCE II 500 MHz NMR spectrometer in DMSO-*d*_6_ supplemented with a trace amount of trifluoroacetic acid using the signals of the residual solvent protons (δ_H_ 2.49) and carbon atoms (δ_C_ 39.5) as internal standards for compounds **1**–**5**, or in CDCl_3_ using the signals of the residual solvent protons (δ_H_ 7.27) and carbon atoms (δ_C_ 77.0) as internal standards for other compounds. HRESITOFMS spectra were recorded on a Bruker micrOTOF focus mass spectrometer. An Agilent HP1200 system equipped with a diode array detector was used for analysis and purification.

### Microorganism

The coral sample *Mycedium* sp. was collected at −15 to −20 m from Tanjung Gelam, Karimunjawa National Park, Jepara, Central Java, Indonesia with a permission number of 1096/T.34/TU/SIMAKSI/7/2017. The strain T35-5 was isolated according to the method described previously [35] and identified as a member of the genus *Kocuria* on the basis of 100.0% similarity in the 16S rRNA gene sequence (1381 nucleotides; DDBJ accession number LC556325) to *Kocuria palustris* DSM 11925^T^ (accession number Y16263).

### Fermentation

In a similar manner as described in [22], the strain T35-5 was maintained on Marine Agar 2216 (Difco). A loopful of the strain T35-5 was inoculated into a 500 mL K-1 flask containing 100 mL of Marine Broth 2216 (Difco) as a seed culture. The seed culture was incubated at 30 °C on a rotary shaker at 200 rpm for 2 days. Three mL each of the seed culture were inoculated into 500 mL K-1 flasks containing 100 mL of A11M production medium, which consists of 0.2% glucose, 2.5% soluble starch, 0.5% yeast extract, 0.5% polypeptone (Wako Pure Chemical Industries, Ltd.), 0.5% NZ-amine (Wako Pure Chemical Industries, Ltd.), 0.3% CaCO_3_, and 1% Diaion HP-20 (Mitsubishi Chemical Co.) in natural seawater (collected from Toyama Bay, Japan). The pH value of the medium was adjusted to 7.0 before sterilization. The inoculated flasks were incubated at 30 °C for 5 days, with rotational shaking at 200 rpm.

### Extraction and isolation

In a similar manner as described in [22], after fermentation, 100 mL of 1-butanol was added to each flask, and the flasks were shaken for 1 h. The emulsified mixture was centrifuged at 6000 rpm for 10 min, and the organic layer was separated from the aqueous layer. Evaporation of the organic solvent gave approximately 3.8 g of extract from 3 L of culture. The extract (3.8 g) was chromatographed over a silica gel column using a mixture solvent of CHCl_3_/MeOH (1:0, 20:1, 10:1, 4:1, 2:1, 1:1, and 0:1, v/v). Fraction 3 (10:1) was concentrated to yield 0.38 g of a brown oil, which was further fractionated by ODS column chromatography with a stepwise gradient of a MeCN/0.1% HCO_2_H aqueous solution (2:8, 3:7, 4:6, 5:5, 6:4, 7:3, and 8:2, v/v). Fractions 4 (5:5) and 5 (6:4) were separately concentrated in vacuo, and the remaining aqueous layer was extracted with EtOAc. The organic layer was dried over anhydrous Na_2_SO_4_, filtered, and concentrated to give 40 mg and 45 mg of a semipure material. Final purification was achieved by preparative HPLC (Cosmosil AR-II, Nacalai Tesque Inc., 10 × 250 mm, 4 mL/min, UV detection at 254 nm) with an isocratic elution of MeCN/0.1% HCO_2_H (42:58) to afford nocarimidazole C (**1**, 2.4 mg, *t*_R_ 8.45 min), nocarimidazole D (**2**, 3.2 mg, *t*_R_ 14.9 min), nocarimidazole A (**3**, 3.8 mg, *t*_R_ 13.5 min), nocarimidazole B (**4**, 8.0 mg, *t*_R_ 17.3 min) from fraction 4, and bulbimidazole A (**5**, 3.2 mg, *t*_R_ 11.2 min) from fraction 5.

### Bioassays

The antimicrobial activity was evaluated in a similar manner as previously reported [22]. The cytotoxicity against P388 murine leukemia cells was examined according to a protocol described in [22].

## Supporting Information

File 1 Copies of the NMR spectra for compounds **1** and **2**.
