# Supplementary material for: Nocarimidazoles C and D, antimicrobial alkanoylimidazoles from a coral-derived actinomycete Kocuria sp.: application of 1JC,H coupling constants for the unequivocal determination of substituted imidazoles and stereochemical diversity of anteisoalkyl chains in microbial metabolites
Source: Beilstein J Org Chem. 2020 Nov 5;16:2719–27. doi: 10.3762/bjoc.16.222 (PMC7653330; doi:10.3762/bjoc.16.222)

BEILSTEIN JOURNAL OF ORGANIC CHEMISTRY

Supporting Information

for

Nocarimidazoles C and D, antimicrobial alkanoylimidazoles from a coral-derived actinomycete *Kocuria* sp.: application of $^{1}J_{C,H}$ coupling constants for the unequivocal determination of substituted imidazoles and stereochemical diversity of anteisoalkyl chains in microbial metabolites

Md. Rokon Ul Karim, Enjuro Harunari, Amit Raj Sharma, Naoya Oku, Kazuaki Akasaka, Daisuke Urabe, Mada Triandala Sibero and Yasuhiro Igarashi

Beilstein J. Org. Chem. **2020,** *16,* 2719–2727. doi:10.3762/bjoc.16.222

Copies of the NMR spectra for compounds 1 and 2

License and Terms: This is a supporting information file under the terms of the Creative Commons Attribution License (https://creativecommons.org/licenses/by/4.0). Please note that the reuse, redistribution and reproduction in particular requires that the authors and source are credited.
The license is subject to the *Beilstein Journal of Organic Chemistry* terms and conditions: (https://www.beilstein-journals.org/bjoc)

Table of Contents

Experimental section S2
Figure S1. UV spectrum of nocarimidazole C (**1**) S6
Figure S2. IR spectrum of **1** S7
Figure S3. ^{1}H NMR spectrum of **1** (500 MHz, DMSO-d_6 with TFA) S8
Figure S4. ^{13}C NMR spectrum of **1** (125 MHz, DMSO-d_6 with TFA) S9
Figure S5. COSY spectrum of **1** (500 MHz, DMSO-d_6 with TFA) S10
Figure S6. HSQC spectrum of **1** (500 MHz, DMSO-d_6 with TFA) S11
Figure S7. Coupled HSQC spectrum of **1** (500 MHz, DMSO-d_6 with TFA) S12
Figure S8. HMBC spectrum of **1** (500 MHz, DMSO-d_6 with TFA) S13
Figure S9. UV spectrum of nocarimidazole D (**2**) S14
Figure S10. IR spectrum of **2** S15
Figure S11. ^{1}H NMR spectrum of **2** (500 MHz, DMSO-d_6 with TFA) S16
Figure S12. ^{13}C NMR spectrum of **2** (125 MHz, DMSO-d_6 with TFA) S17
Figure S13. COSY spectrum of **2** (500 MHz, DMSO-d_6 with TFA) S18
Figure S14. HSQC spectrum of **2** (500 MHz, DMSO-d_6 with TFA) S19
Figure S15. HMBC spectrum of **2** (500 MHz, DMSO-d_6 with TFA) S20
Figure S16. ^{1}H NMR spectrum of **3** (500 MHz, DMSO-d_6 with TFA) S21
Figure S17. ^{13}C NMR spectrum of **3** (125 MHz, DMSO-d_6 with TFA) S22
Figure S18. ^{1}H NMR spectrum of **4** (500 MHz, DMSO-d_6 with TFA) S23
Figure S19. ^{13}C NMR spectrum of **4** (125 MHz, DMSO-d_6 with TFA) S24
Figure S20. ^{1}H NMR spectrum of **5** (500 MHz, DMSO-d_6 with TFA) S25
Figure S21. ^{13}C NMR spectrum of **5** (125 MHz, DMSO-d_6 with TFA) S26
Figure S22. Coupled HSQC spectrum of **5** (500 MHz, DMSO-d_6 with TFA) S27
Figure S23. ^{1}H NMR spectrum of **8** (500 MHz, DMSO-d_6 with TFA) S28
Figure S24. ^{13}C NMR spectrum of **8** (125 MHz, DMSO-d_6 with TFA) S29
Figure S25. HSQC spectrum of **8** (500 MHz, DMSO-d_6 with TFA) S30
Figure S26. Coupled HSQC spectrum of **8** (500 MHz, DMSO-d_6 with TFA) S31
Figure S27. HMBC spectrum of **8** (500 MHz, DMSO-d_6 with TFA) S32
Figure S28. ^{1}H NMR spectrum of **9** (500 MHz, DMSO-d_6 with TFA) S33
Figure S29. ^{13}C NMR spectrum of **9** (125 MHz, DMSO-d_6 with TFA) S34
Figure S30. HSQC spectrum of **9** (500 MHz, DMSO-d_6 with TFA) S35
Figure S31. Coupled HSQC spectrum of **9** (500 MHz, DMSO-d_6 with TFA) S36
Figure S32. HMBC spectrum of **9** (500 MHz, DMSO-d_6 with TFA) S37
Figure S33. ^{1}H NMR spectrum of *nat*-**10**-(*R*)-2A1P (500 MHz, $CDCl_3$) S38
Figure S34. ^{1}H NMR spectrum of authentic (*S*)-**10**-(*R*)-2A1P (500 MHz, $CDCl_3$) S39
Figure S35. ^{1}H NMR spectrum of authentic (*S*)-**10**-(*S*)-2A1P (500 MHz, $CDCl_3$) S40
Figure S36. ^{1}H NMR spectrum of *nat*-**11**-(*R*)-2A1P (500 MHz, $CDCl_3$) S41
Figure S37. ^{1}H NMR spectrum of *nat*-**12**-(*R*)-2A1P (500 MHz, $CDCl_3$) S42
Figure S38. ^{1}H NMR spectrum of authentic (*S*)-**11**-(*R*)-2A1P (500 MHz, $CDCl_3$) S43
Figure S39. ^{1}H NMR spectrum of authentic (*S*)-**11**-(*S*)-2A1P (500 MHz, $CDCl_3$) S44

Experimental section

Nocarimidazole C (**1**): pale yellow amorphous solid; $[\alpha]^{23}_{D}$ -1.2 (*c* 0.10, MeOH); UV (MeOH) λ_{max} (log ε) 296 (4.27), 226 (3.87), 200 (4.30) nm; IR (ATR) ν_{max} 3127, 2955, 2923, 1664 cm^{-1}; ^{1}H and ^{13}C NMR data, see Table 1; HRESITOFMS *m/z* 224.1763 [M + H]$^{+}$ (calcd for $C_{12}H_{22}N_3O$, 224.1757).

Nocarimidazole D (**2**): pale yellow amorphous solid; UV (MeOH) λ_{max} (log ε) 296 (4.47), 225 (4.07), 200 (4.52) nm; IR (ATR) ν_{max} 3126, 2954, 2923, 1667 cm^{-1}; ^{1}H and ^{13}C NMR data, see Table 1; HRESITOFMS *m/z* 238.1915 [M + H]$^{+}$ (calcd for $C_{13}H_{24}N_3O$, 238.1913).

Nocarimidazole A (**3**): UV (MeOH) λ_{max} (log ε) 295 (4.35), 232 (3.91), 200 (4.36) nm; HRESITOFMS *m/z* 238.1910 [M + H]$^{+}$ (calcd for $C_{13}H_{24}N_3O$, 238.1914); ^{1}H and ^{13}C NMR data in $CDCl_3$ was the same as those reported in Reference 21 from the Main Manuscript.

Nocarimidazole B (**4**): $[\alpha]^{23}_{D}$ +1.2 (*c* 0.10, MeOH); UV (MeOH) λ_{max} (log ε) 296 (4.32), 233 (3.89), 200 (4.34) nm; HRESITOFMS *m/z* 252.2074 [M + H]$^{+}$ (calcd for $C_{14}H_{26}N_3O$, 252.2070); ^{1}H and ^{13}C NMR data in $CDCl_3$ was the same as those reported in Reference 21 from the Main Manuscript.

Bulbimidazole A (**5**): $[\alpha]^{23}_{D}$ +1.2 (*c* 0.10, MeOH); HRESITOFMS *m/z* 237.1965 [M + H]$^{+}$ (calcd for $C_{14}H_{25}N_2O$, 237.1961); ^{1}H and ^{13}C NMR data in DMSO-d_6 with a trace amount of TFA was the same as those reported in Reference 22 from the Main Manuscript.

Synthesis of 1-(5-amino-1*H*-imidazol-4-yl)ethan-1-one (8)

To a solution of 4-isocyano-1*H*-imidazol-5-amine (100 mg, 92 μmol) in THF (9.2 mL) was added 1.0 M solution of MeMgBr (4.6 mL) at room temperature. After stirring for 2 h, 3 M HCl (10 mL) was added to the reaction mixture. After stirring at 90 °C for 2 h, the reaction mixture was cooled to the ambient temperature and concentrated in vacuo. The resulting liquid mixture received a saturated solution of $NaHCO_3$ (100 mL) and was then extracted with EtOAc. The organic layer was then washed with water and brine, dried over anhydrous Na_2SO_4 and concentrated in vacuo to give

62 mg of the crude material. This was then purified by preparative HPLC (XTerra Shield RP18 OBD™ Prep Column, 10 μm, 10 × 250 mm, 4 mL/min, Waters) with an isocratic elution MeCN/10 mM NH_4HCO_3 (5:95) to afford 1-(5-amino-1*H*-imidazol-4-yl)ethan-1-one (**8**, 32 mg, 27% yield): ^1H NMR (DMSO-d_6 with TFA, 500 MHz) δ_H 2.27 (3H, s), 7.77 (1H, s); ^{13}C NMR (DMSO-d_6 with TFA, 125 MHz) δ_C 187.1, 148.9, 132.4, 113.4, 26.3; HRESITOFMS *m/z* 126.0665 $[M + H]^+$ (calcd for $C_5H_8N_3O$ 126.0667).

Synthesis of 1-(2-amino-1*H*-imidazol-4-yl)ethan-1-one (9)

To a solution of pyrimidin-2-amine (100 mg, 105 μmol) in toluene (4 mL) was added 1,1-dimethoxy-*N*,*N*-dimethylmethanamine (200 μL, 157 μmol), and the mixture was stirred at 110 °C for 2 h. After cooling, EtOAc was added to the reaction mixture, and this organic layer was washed with water and brine, dried over anhydrous Na_2SO_4 and concentrated in vacuo to afford (*E*)-*N*,*N*-dimethyl-*N*'-(pyrimidin-2-yl)formimidamide (88 mg).

(*E*)-*N*,*N*-Dimethyl-*N*'-(pyrimidin-2-yl)formimidamide (88 mg, 59 μmol) was then treated with 1-chloropropan-2-one (190 μL, 210 μmol) in dry CH_2Cl_2 (4 mL) at room temperature. After stirring for 2 days, EtOAc was added to the reaction mixture, and the organic layer was washed with water and brine, dried over anhydrous Na_2SO_4 and concentrated under reduced pressure to give 3-acetylimidazo[1,2-a]pyrimidine (82 mg).

A solution of 3-acetylimidazo[1,2-a]pyrimidine (82 mg, 50 μmol) was reacted with N_2H_4 (180 μL, 200 μmol) in H_2O (3 mL) at room temperature for 30 min. The reaction mixture was extracted with EtOAc and the organic layer was washed with water and brine, dried over anhydrous Na_2SO_4 and concentrated in vacuo to give 78 mg of crude product. The crude material was subjected to preparative HPLC (XTerra Shield RP18 OBD™ Prep Column, 10 μm, 10 × 250 mm, 4 mL/min, Waters, MA) with an isocratic elution MeCN/10 mM NH_4HCO_3 (2:98) to yield 1-(5-amino-1*H*-imidazol-4-yl)ethan-1-one (**9**, 65 mg, 56% overall yield): ^1H NMR (DMSO-d_6 with

TFA, 500 MHz) δ_H 2.34 (3H, s), 8.0 (1H, s); ^{13}C NMR (DMSO-d_6 with TFA, 125 MHz) δ_C 186.6, 148.8, 127.0, 122.8, 25.4; HRESITOFMS *m/z* 126.0663 $[M + H]^+$ (calcd for $C_5H_8N_3O$ 126.0667).

Determination of the absolute configuration of 1, 4, and 5 by the Ohrui–Akasaka method

Authentic samples for HPLC comparison of the (*R*)- and (*S*)-2-(anthracene-2,3-dicarboximido)propyl esters of (*S*)-6-methyloctanoic acid ((*S*)-**10**-(*R*)-2A1P and (*S*)-**10**-(*S*)-2A1P) and (*S*)-8-methyldecanoic acid ((*S*)-**11**-(*R*)-2A1P and (*S*)-**11**-(*S*)-2A1P) were prepared as described in our previous study, see References 22 and 30 from the Main Manuscript.

Nocarimidazole C (**1**, 0.5 mg, 2 μmol) was converted to the ester derivative *nat*-**10**-(*R*)-2A1P (1.1 mg) in a similar manner as described previously in Reference 22 from the Main Manuscript. Oxidative degradation of nocarimidazole D (**4**, 0.5 mg, 2 μmol) and bulbimidazole A (**5**, 0.5 mg, 2 μmol) and derivatization with (*R*)-2A1P were carried out in a similar manner, yielding the ester derivatives *nat*-**11**-(*R*)-2A1P (0.7 mg) and *nat*-**12**-(*R*)-2A1P (0.7 mg).

nat-**10**-(*R*)-2A1P: HRESITOFMS *m/z* 468.2152 $[M + Na]^+$ (calcd for $C_{28}H_{31}NO_4Na$, 468.2145).

nat-**11**-(*R*)-2A1P: HRESITOFMS *m/z* 496.2455 $[M + Na]^+$ (calcd for $C_{30}H_{35}NO_4Na$, 496.2458).

nat-**12**-(*R*)-2A1P: HRESITOFMS *m/z* 496.2456 $[M + Na]^+$ (calcd for $C_{30}H_{35}NO_4Na$, 496.2458).

nat-**10**-(*R*)-2A1P and synthetic (*S*)-**10**-(*R*)-2A1P and (*S*)-**10**-(*S*)-2A1P were analyzed by HPLC according to the reported protocol in Reference 22 from the Main Manuscript, with minor modifications of the conditions. Column chromatography: tandemly connected Develosil ODS-HG-3 (3.0 mm i. d. × 250 mm + 3.0 mm i. d. × 150 mm, Nomura Chemical); mobile phase: MeCN/MeOH/THF 3:1:1; column temperature: −48.0 °C; flow rate: 0.075 mL/min. The column was cooled by using Cryocool CC-100 (Neslab Instruments Inc.) HPLC peaks were detected by monitoring the fluorescence intensity at 460 nm, with excitation at 298 nm on an FP-4025 fluorescence detector (JASCO Corporation, Hachioji, Japan). The retention times were 177 min for

(*S*)-**10**-(*S*)-2A1P and 184 min for (*S*)-**10**-(*R*)-2A1P, while *nat*-**10**-(*R*)-2A1P gave peaks at 177 and 184 min with an area ratio of 72.9:27.1.

nat-**11**-(*R*)-2A1P derived from nocarimidazoles B (**4**) and synthetic (*S*)-**11**-(*R*)-2A1P and (*S*)-**11**-(*S*)-2A1P were analyzed by HPLC using the same column and solvent system. The column temperature was set at −42.5 °C and the flow rate at 0.10 mL/min. The retention times were 234 min for (*S*)-**11**-(*S*)-2A1P and 244 min for (*S*)-**11**-(*R*)-2A1P, whereas *nat*-**4**-(*R*)-2A1P gave a peak only at 244 min.

nat-**12**-(*R*)-2A1P derived from bulbimidazole A (**5**) and synthetic (*S*)-**11**-(*R*)-2A1P and (*S*)-**11**-(*S*)-2A1P were analyzed by HPLC using the same column and solvent system. The column temperature was set at −42.5 °C and the flow rate at 0.075 mL/min. The retention times were 312 min for (*S*)-**11**-(*S*)-2A1P and 324 min for (*S*)-**11**-(*R*)-2A1P, while *nat*-**12**-(*R*)-2A1P gave peaks at 312 and 324 min in a ratio of 1.4:98.6.

Figure S1. UV spectrum of nocarimidazole C (**1**)

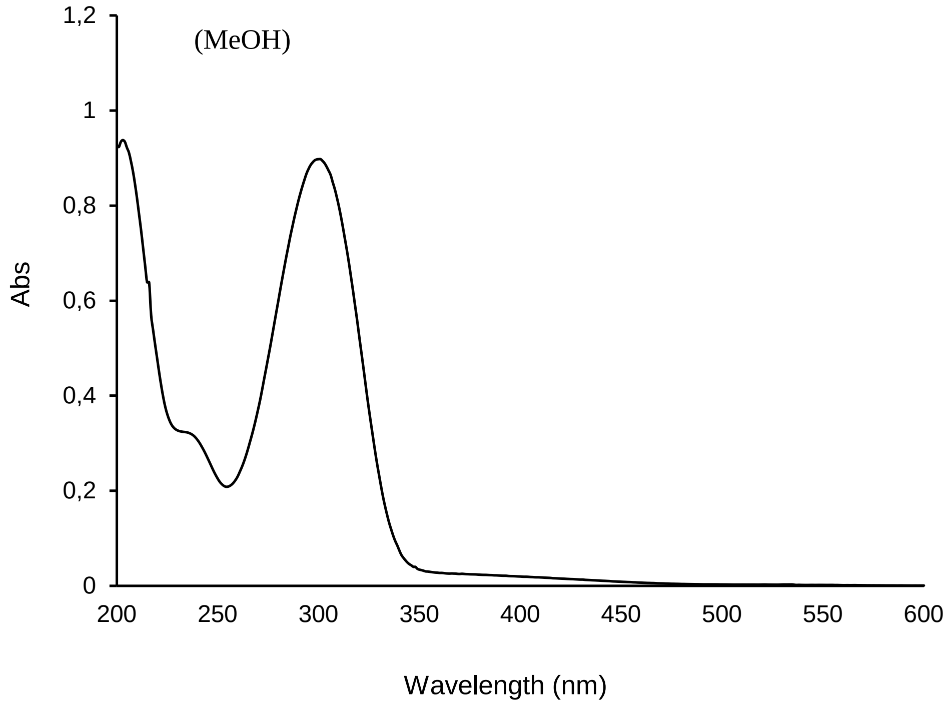

Figure S2. IR spectrum of **1**

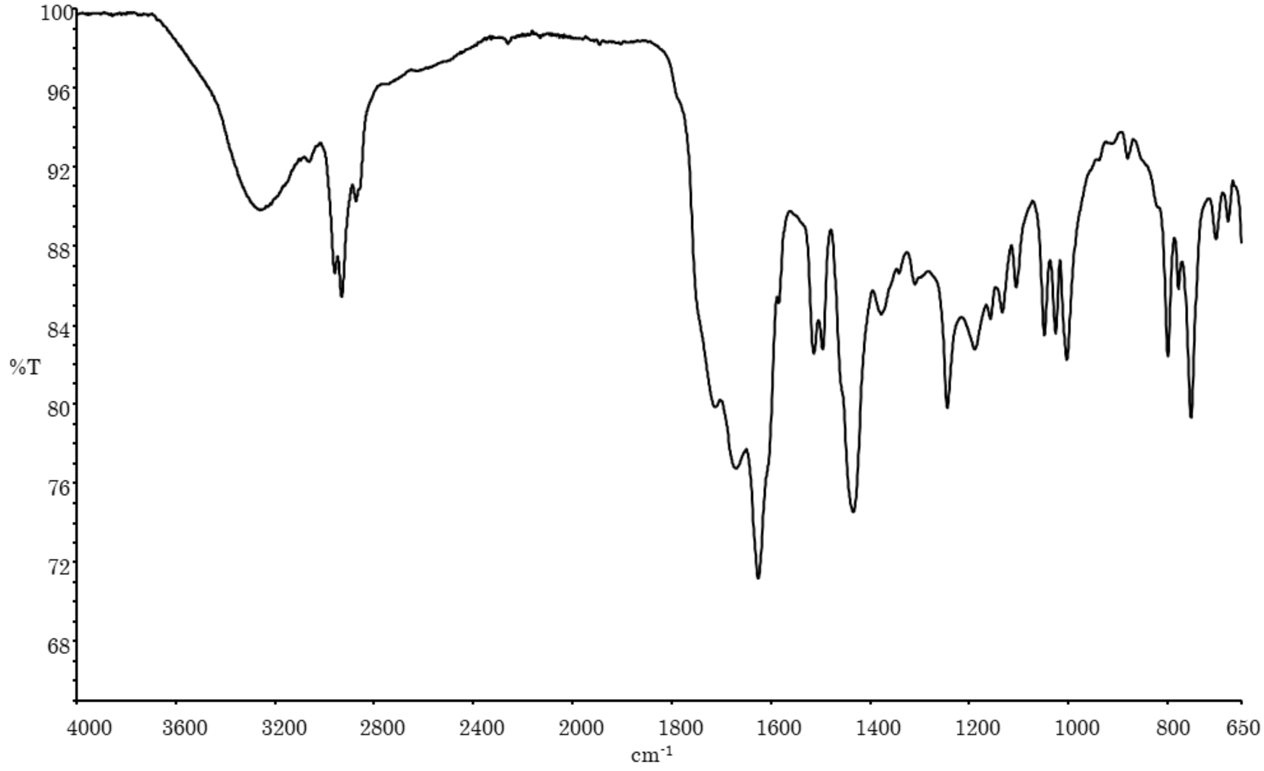

Figure S3. ^{1}H NMR spectrum of **1** (500 MHz, DMSO-d_6 with TFA)

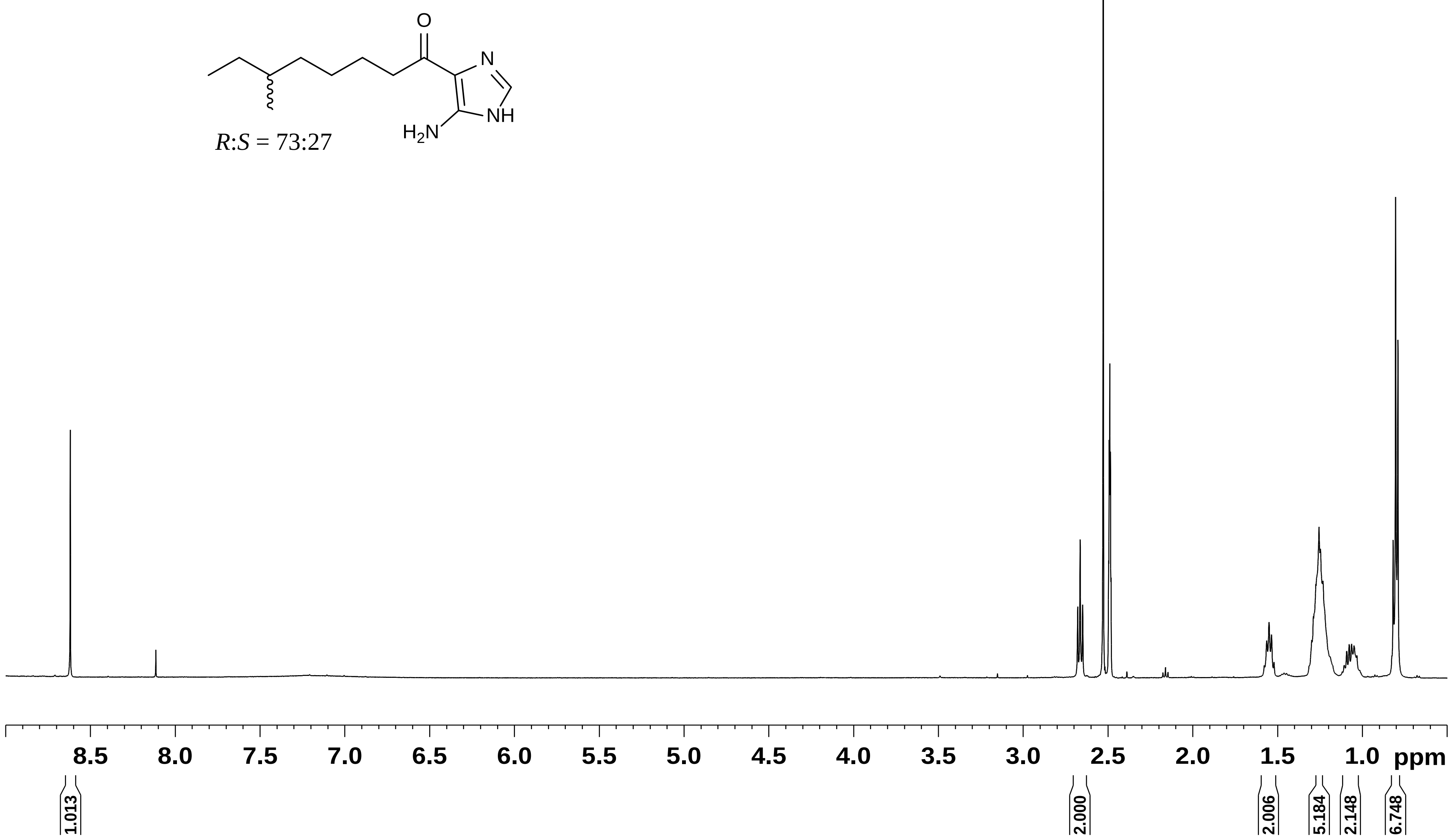

Figure S4. ^{13}C NMR spectrum of **1** (125 MHz, DMSO-d_6 with TFA)

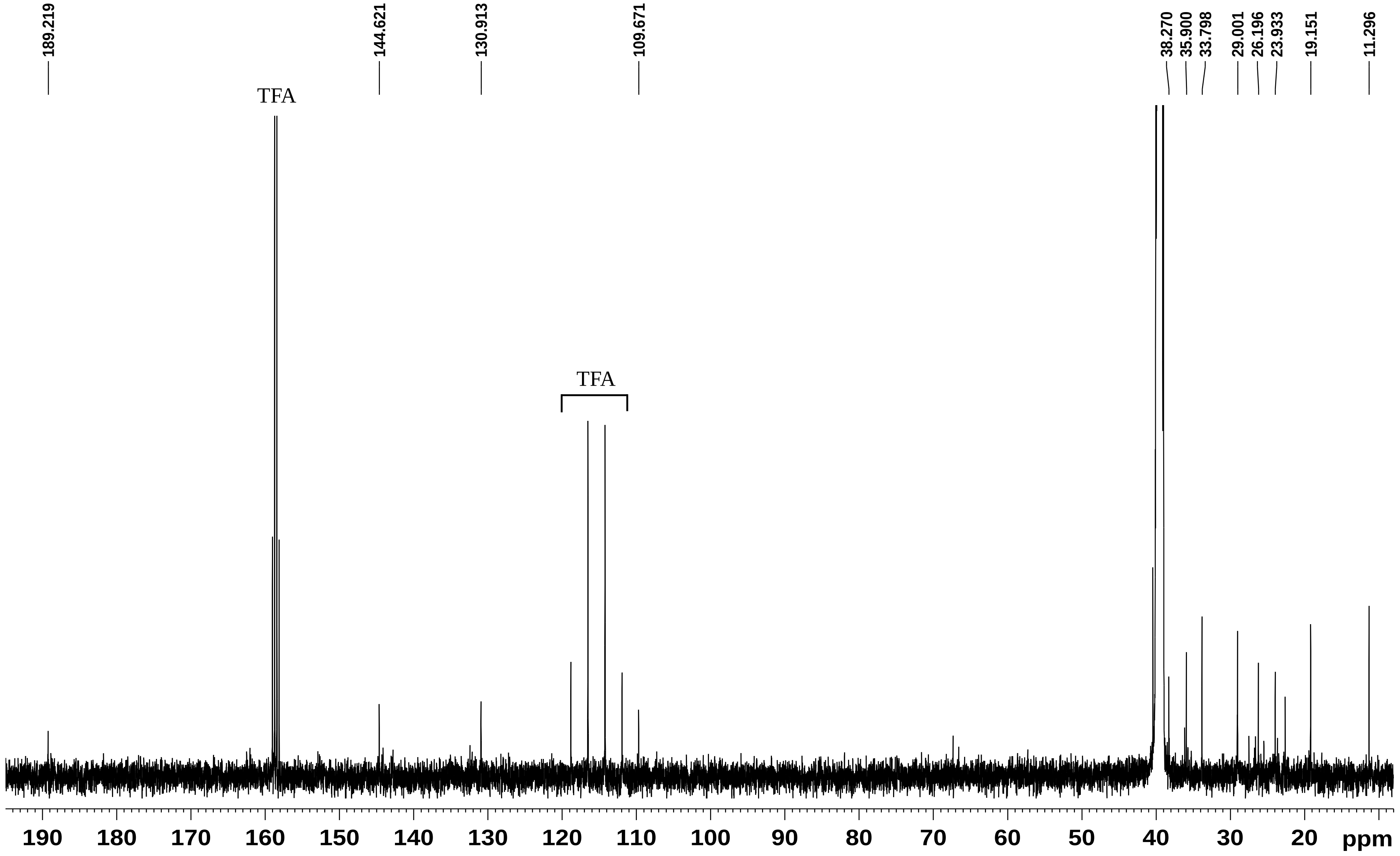

Figure S5. COSY spectrum of **1** (500 MHz, DMSO-d_6 with TFA)

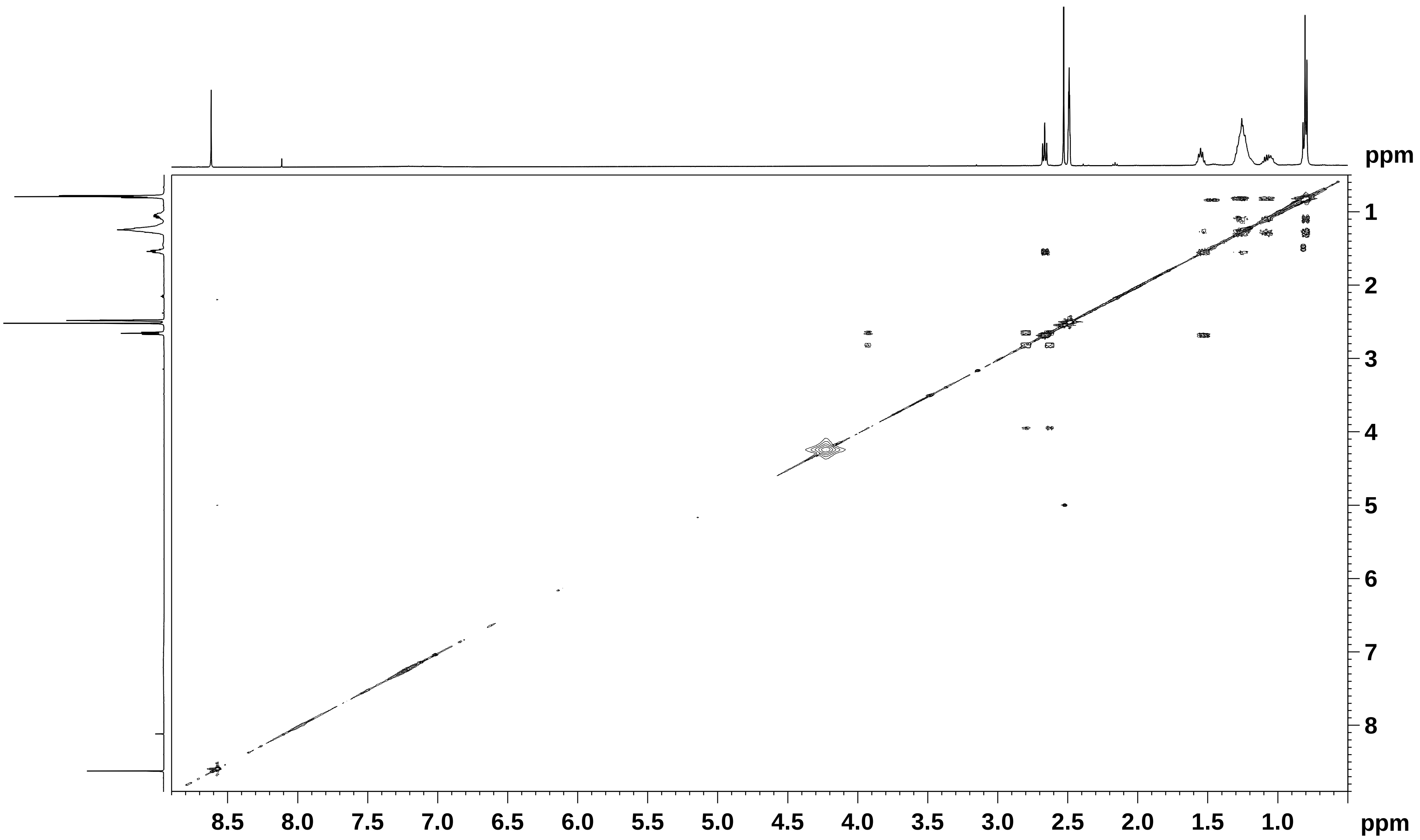

Figure S6. HSQC spectrum of **1** (500 MHz, DMSO-d_6 with TFA)

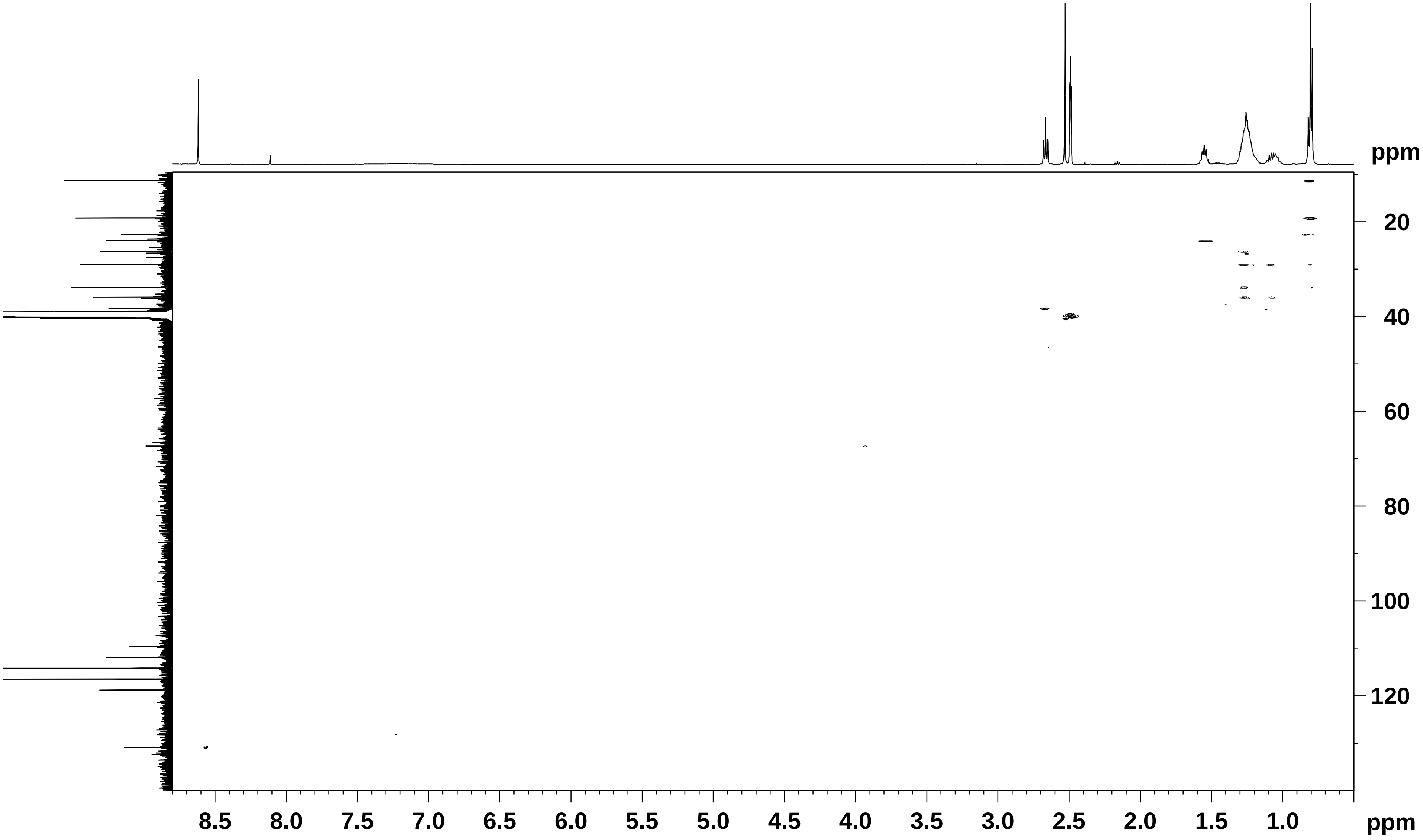

Figure S7. Coupled HSQC spectrum of **1** (500 MHz, DMSO-d_6 with TFA)

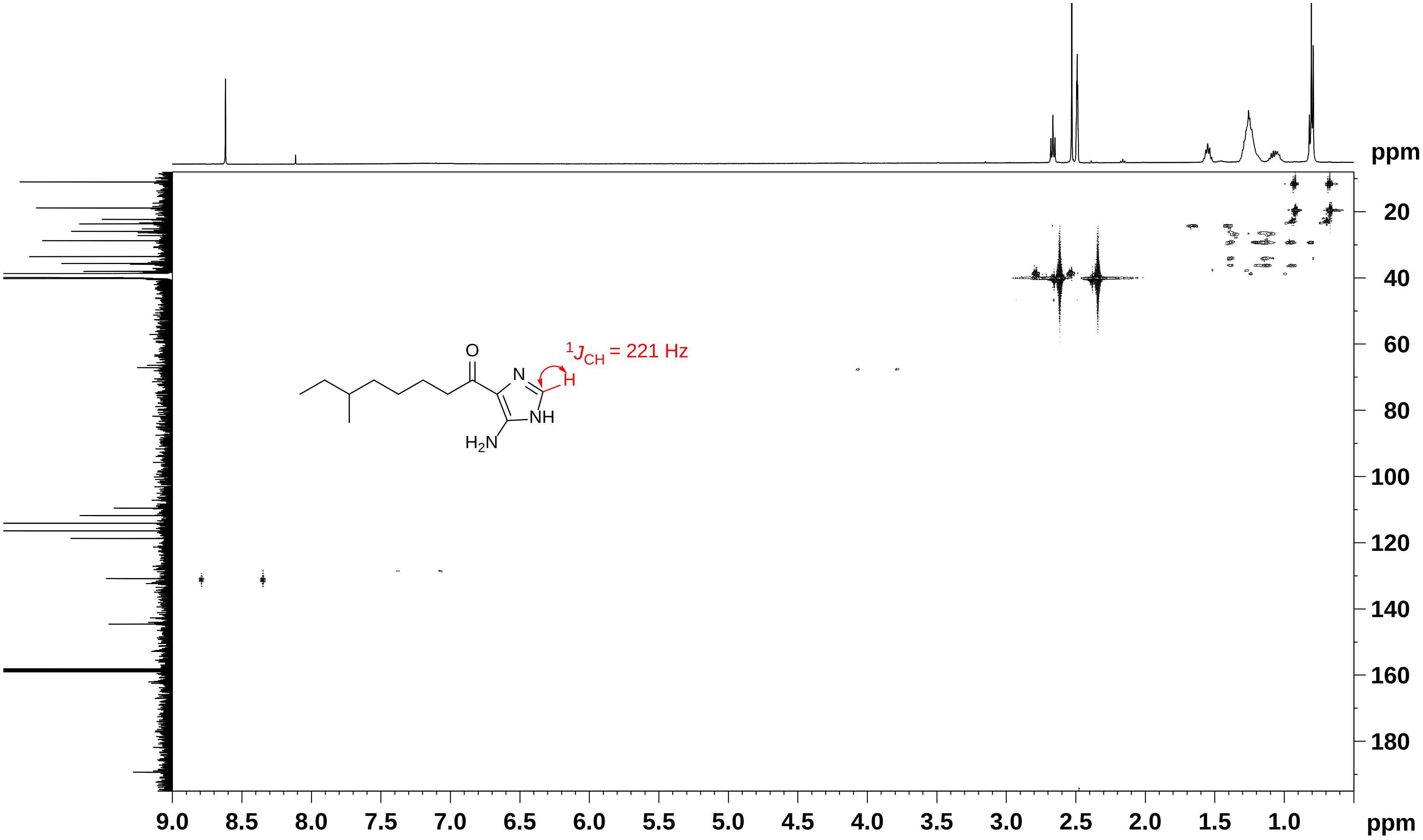

Figure S8. HMBC spectrum of **1** (500 MHz, DMSO-d_6 with TFA)

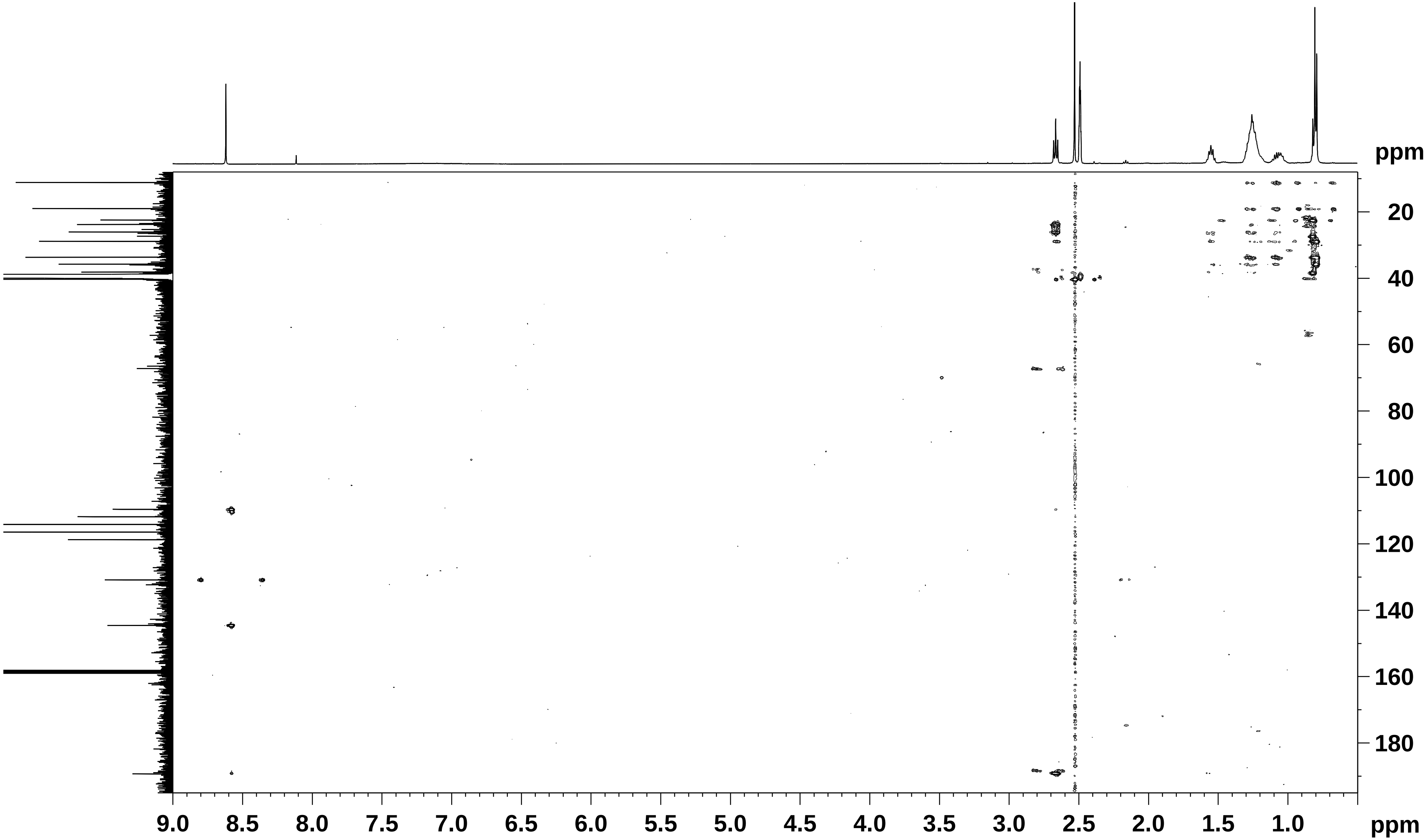

Figure S9. UV spectrum of nocarimidazole D (**2**)

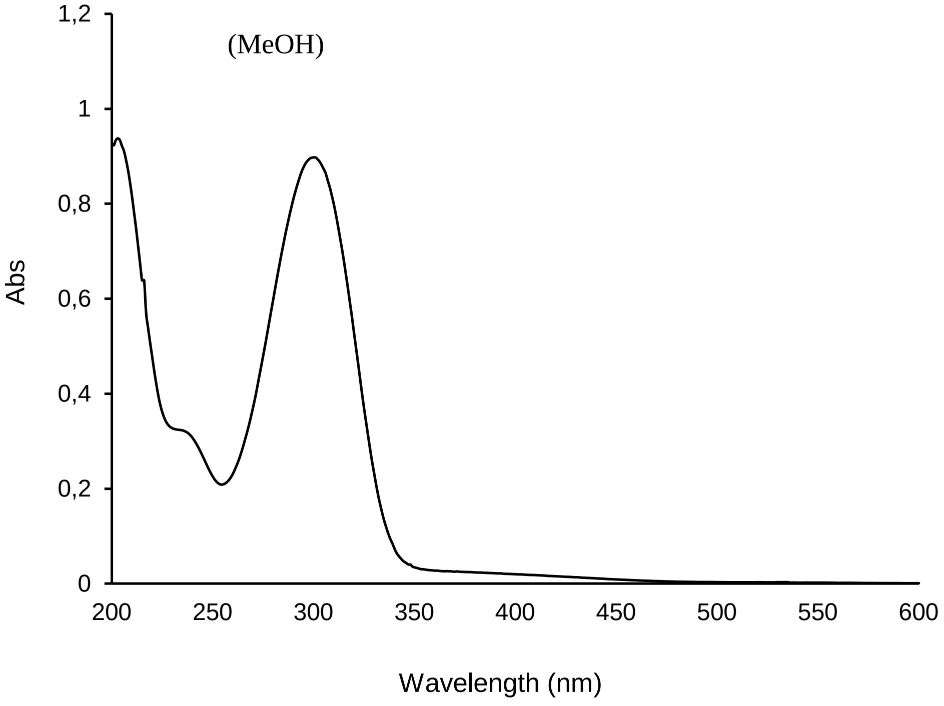

Figure S10. IR spectrum of **2**

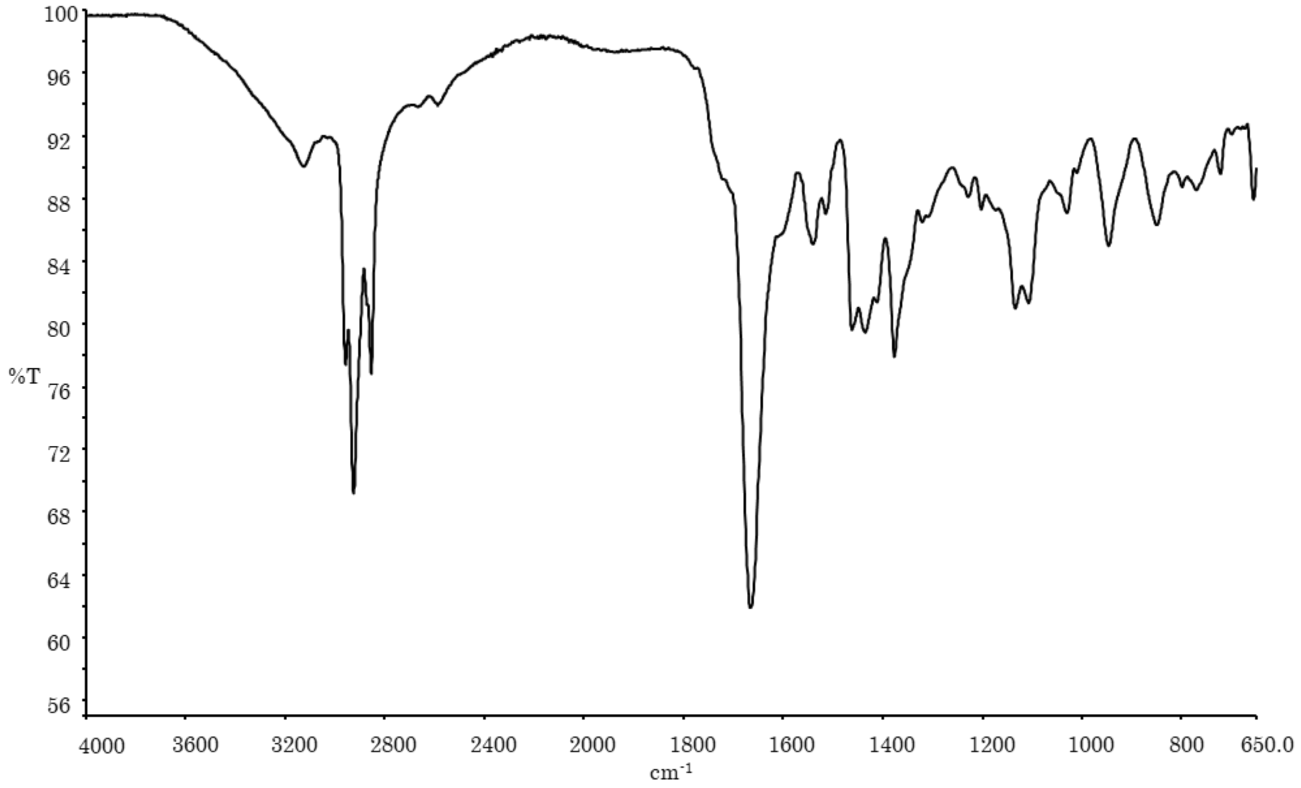

Figure S11. ^{1}H NMR spectrum of **2** (500 MHz, DMSO-d_6 with TFA)

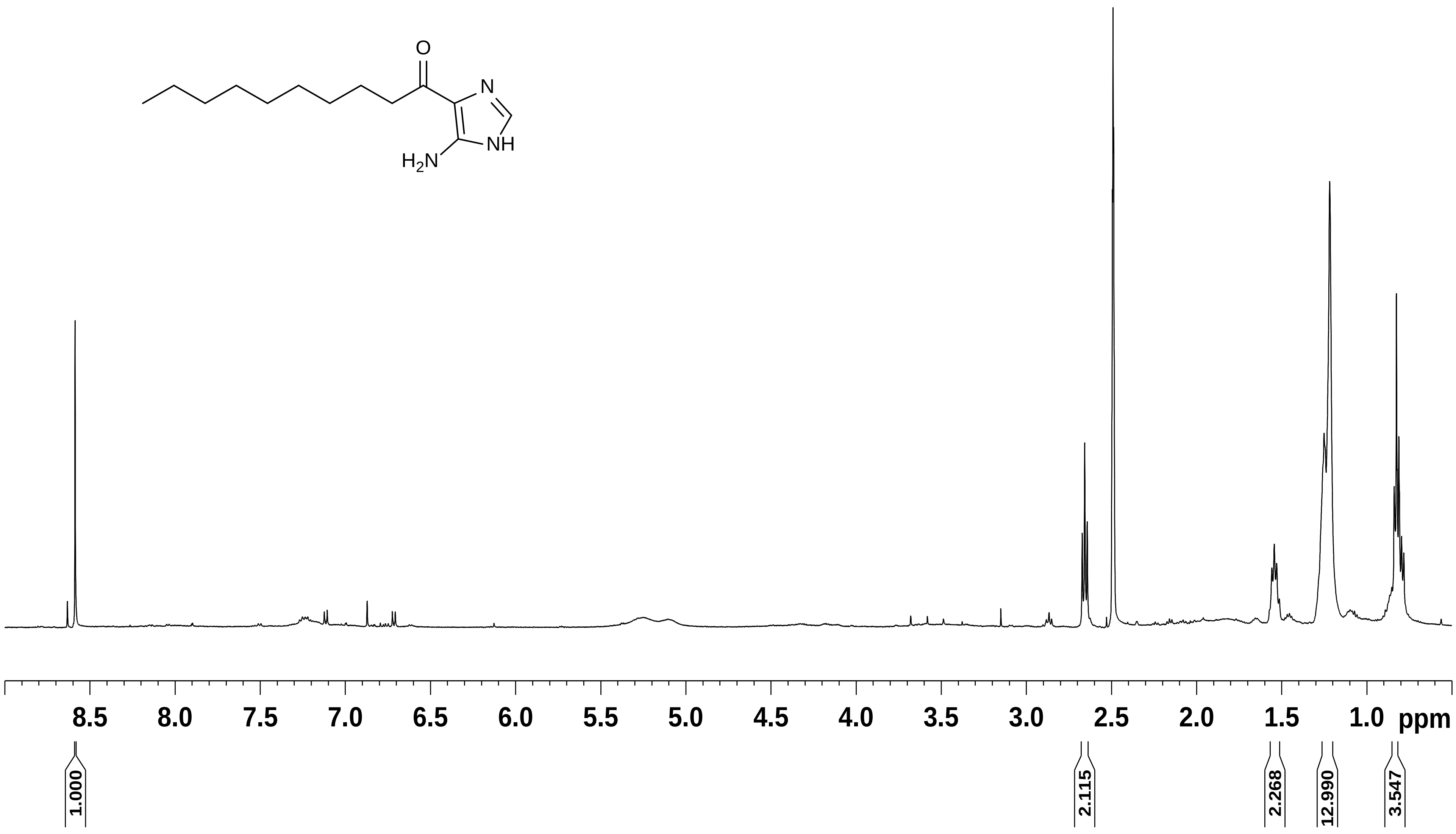

Figure S12. ^{13}C NMR spectrum of **2** (125 MHz, DMSO-d_6 with TFA)

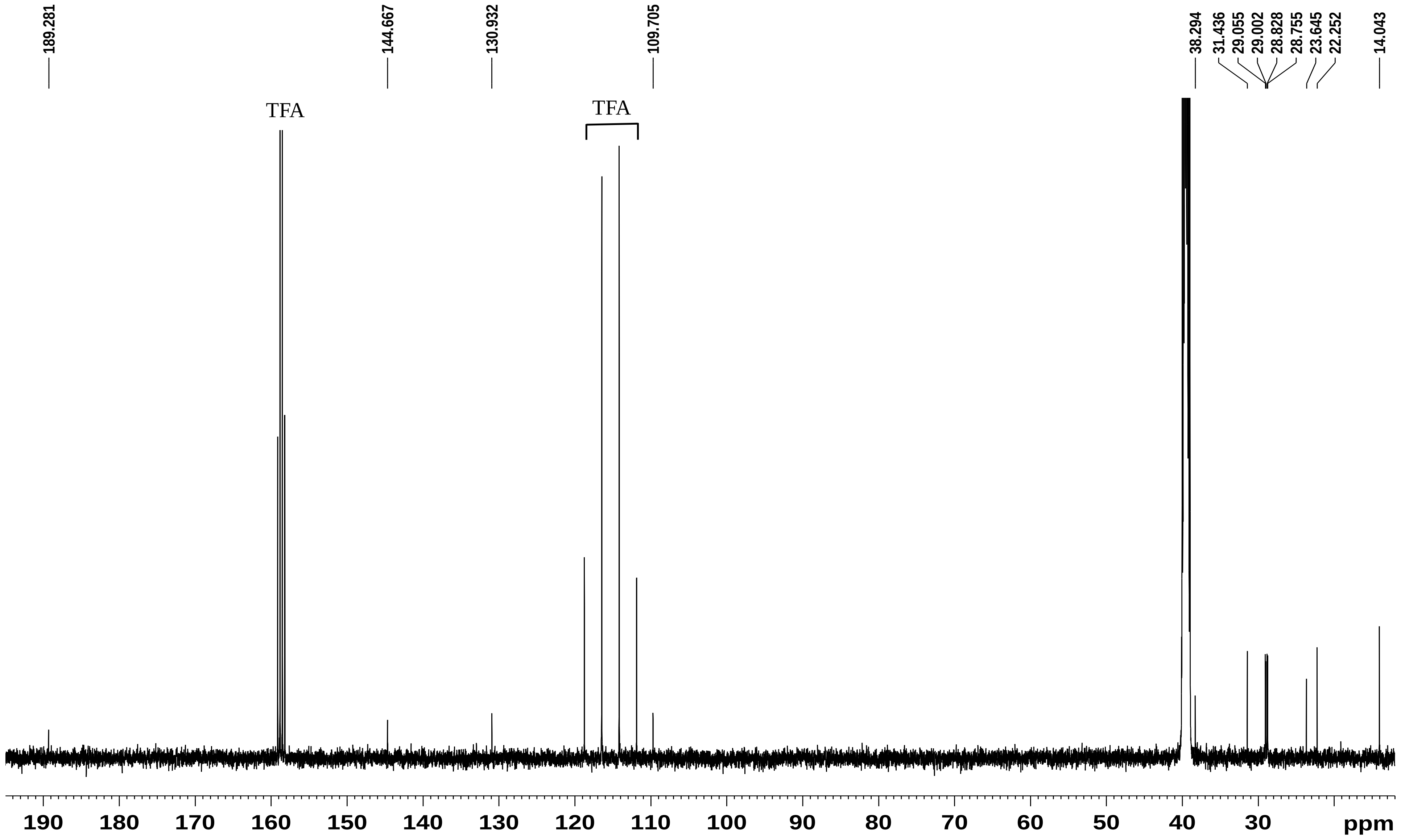

Figure S13. COSY spectrum of **2** (500 MHz, DMSO-d_6 with TFA)

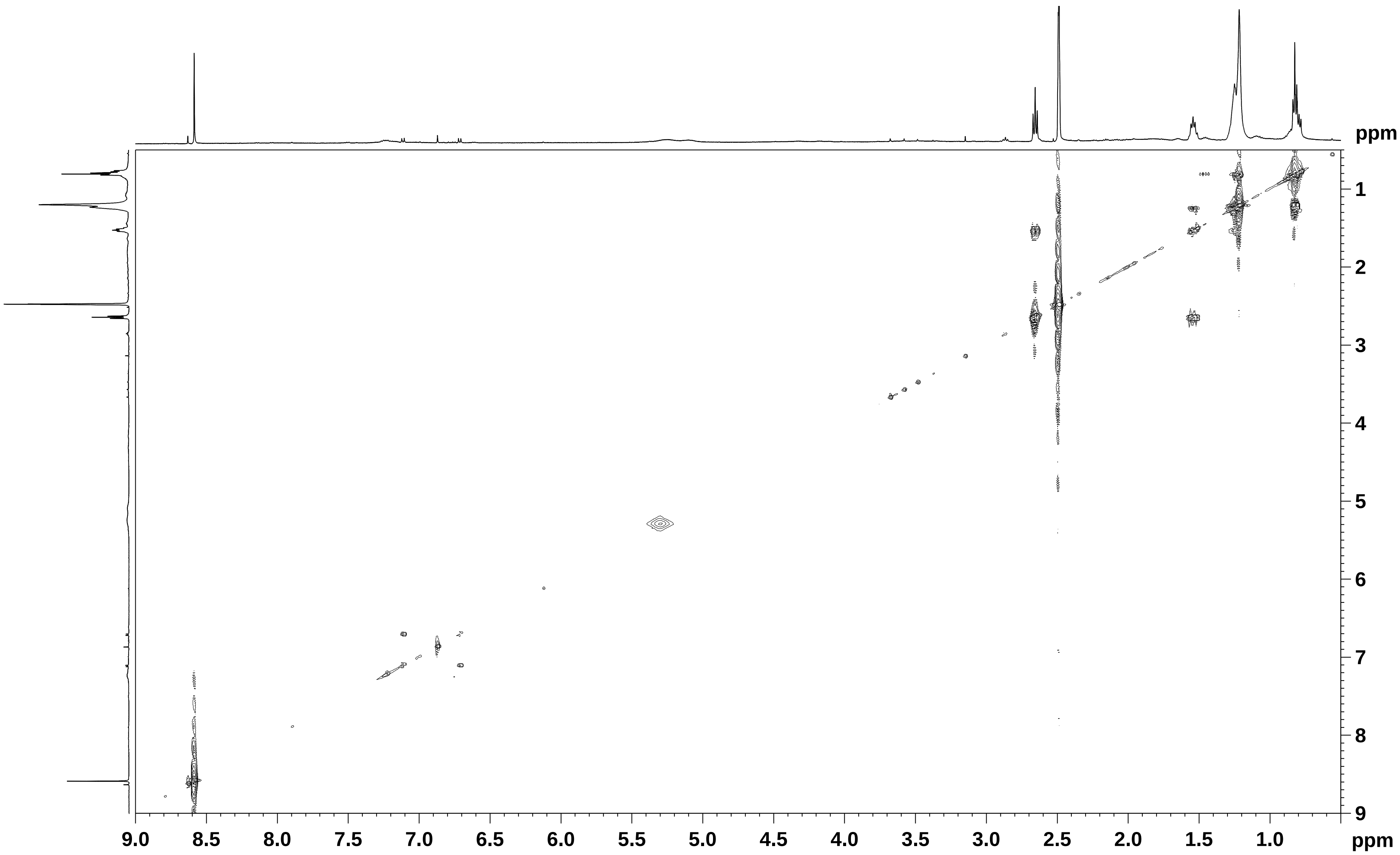

Figure S14. HSQC spectrum of **2** (500 MHz, DMSO-d_6 with TFA

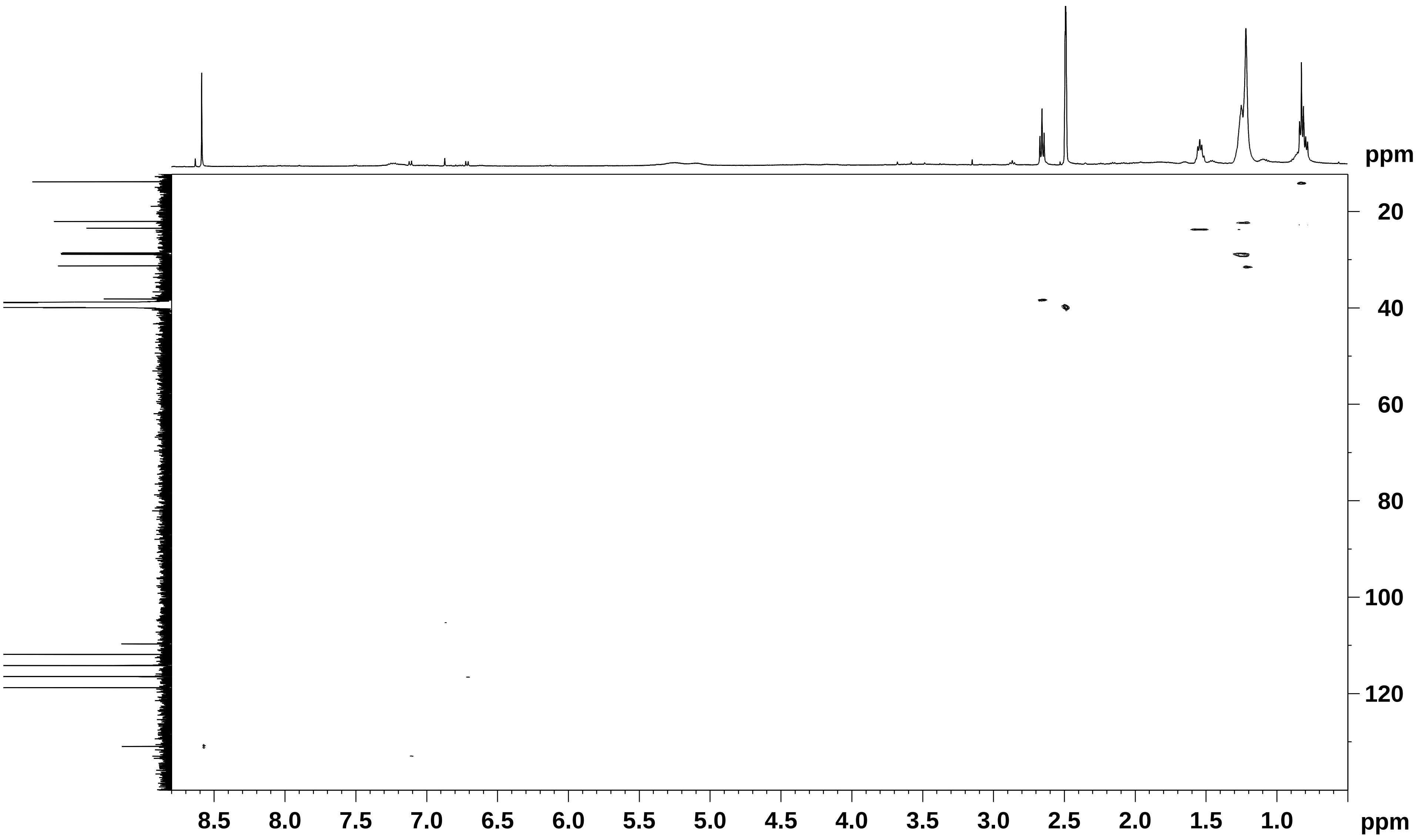

Figure S15. HMBC spectrum of **2** (500 MHz, DMSO-d_6 with TFA)

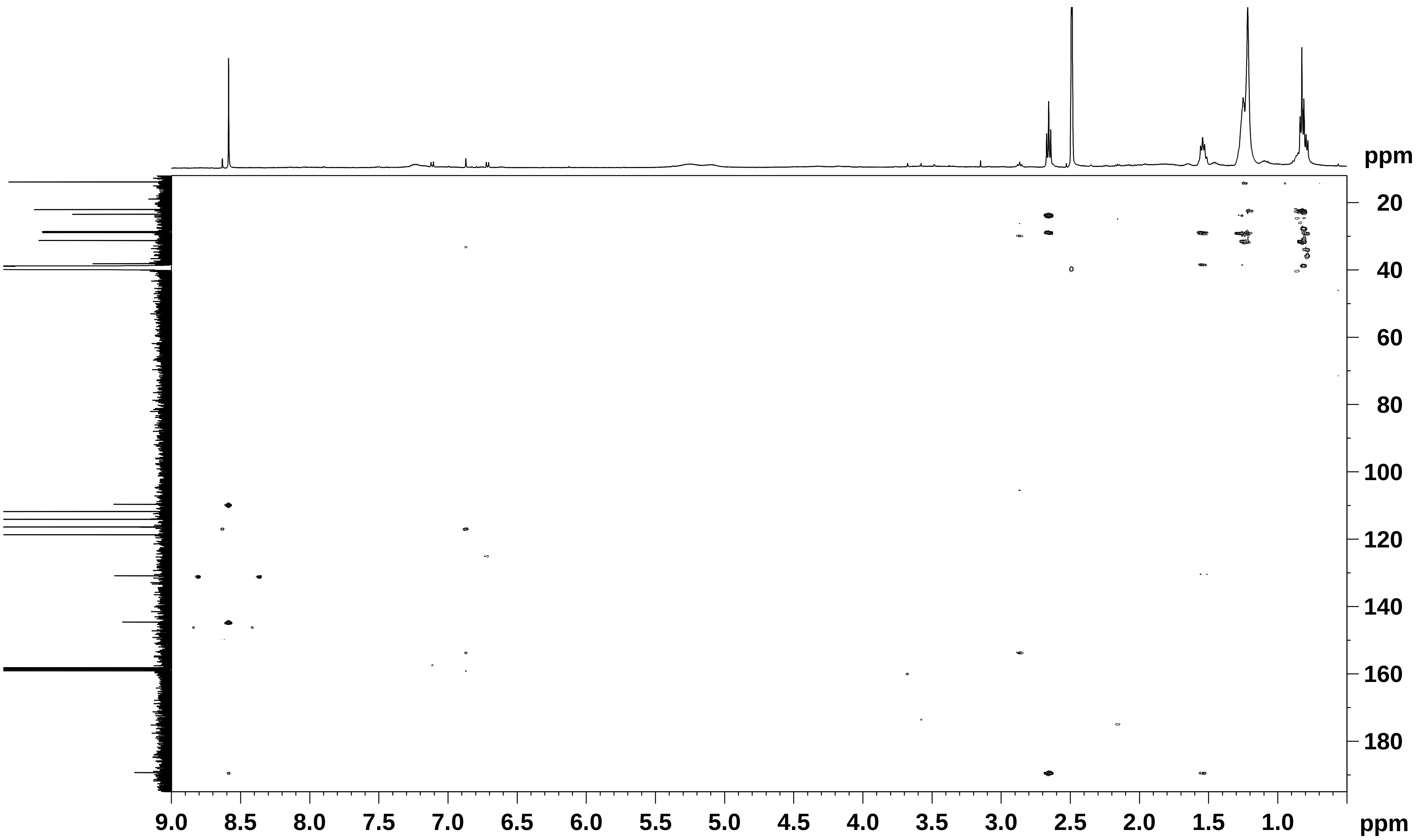

Figure S16. ^{1}H NMR spectrum of **3** (500 MHz, DMSO-d_6 with TFA)

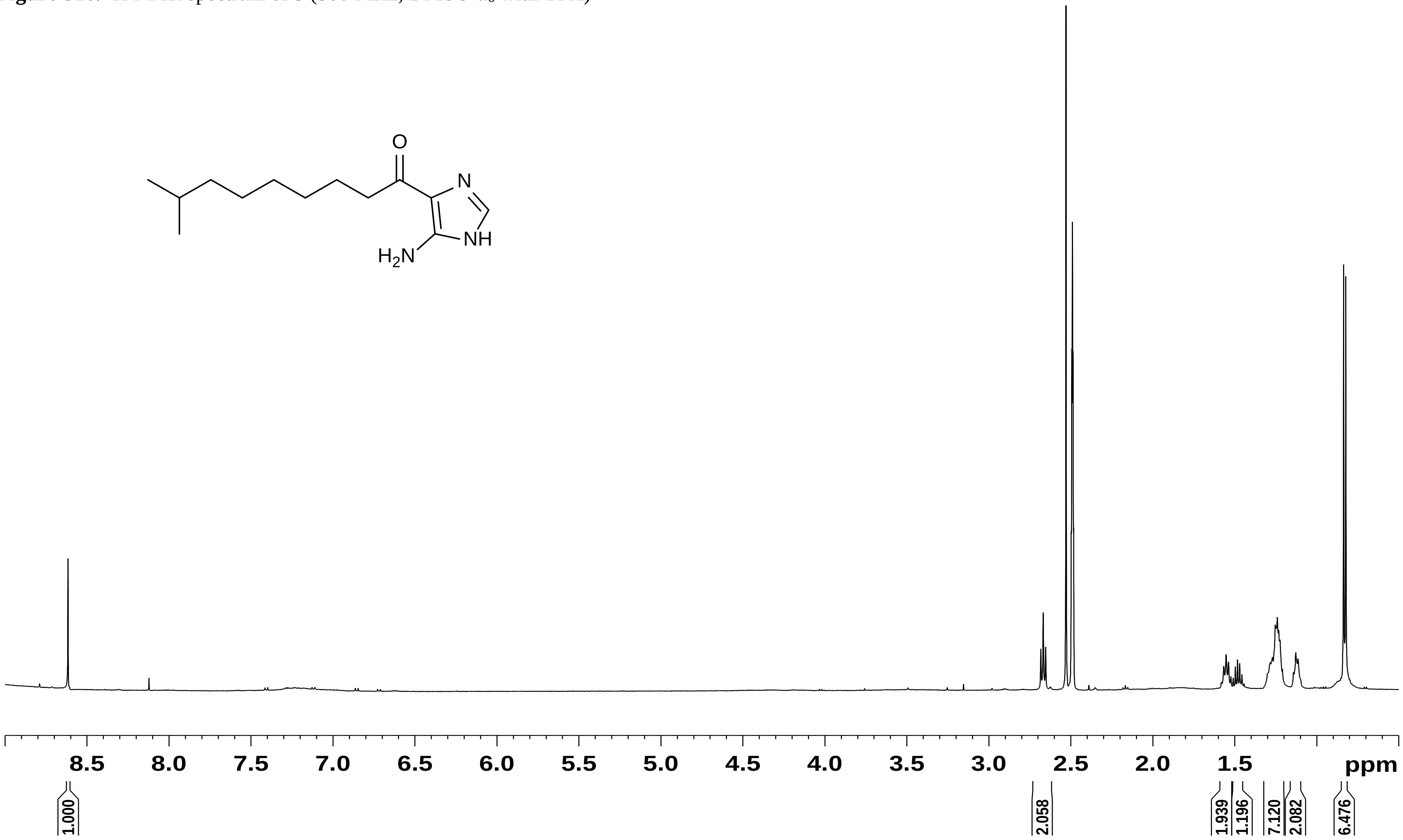

Figure S17. ^{13}C NMR spectrum of **3** (125 MHz, DMSO-d_6 with TFA)

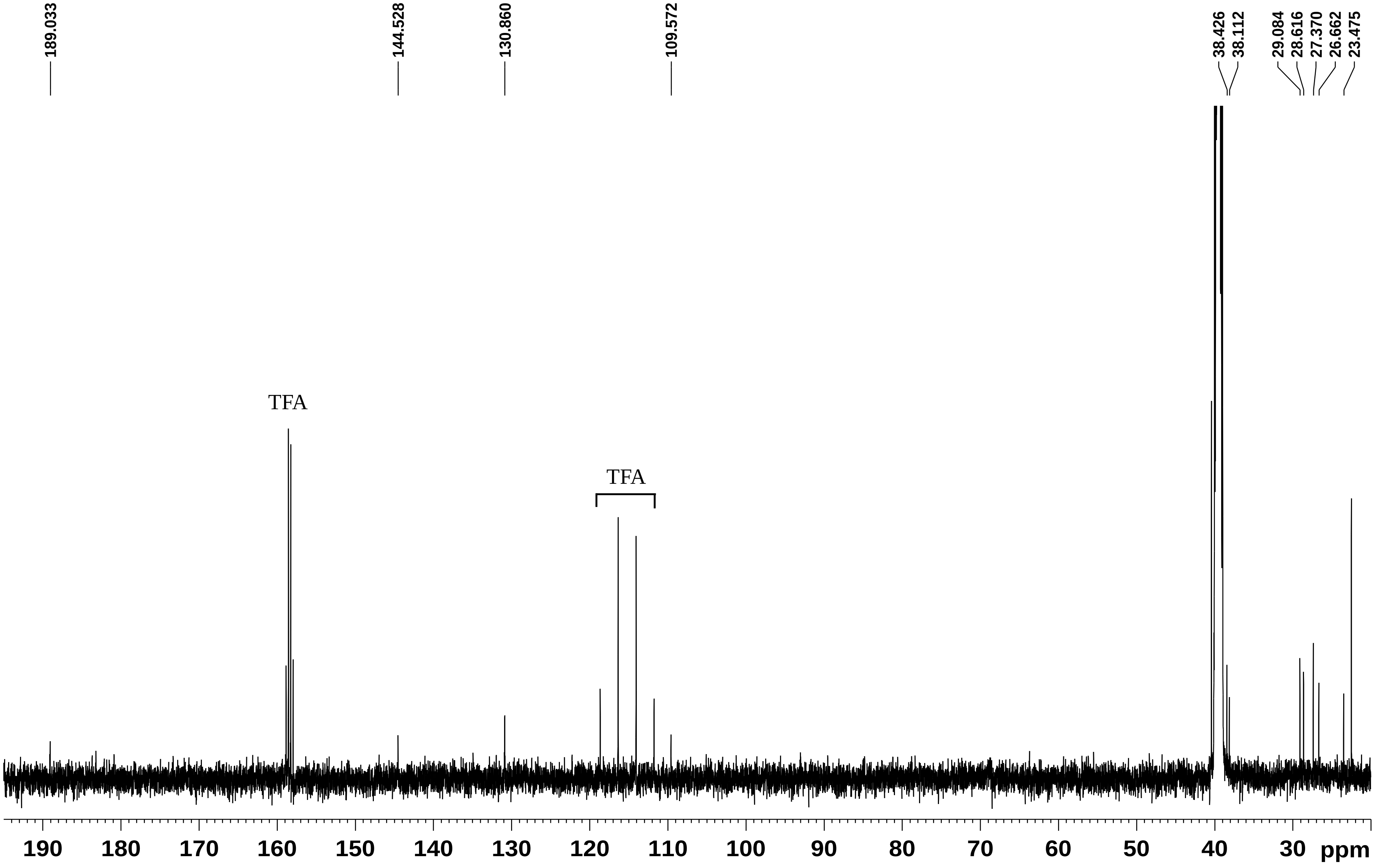

Figure S18. ^{1}H NMR spectrum of **4** (500 MHz, DMSO-d_6 with TFA)

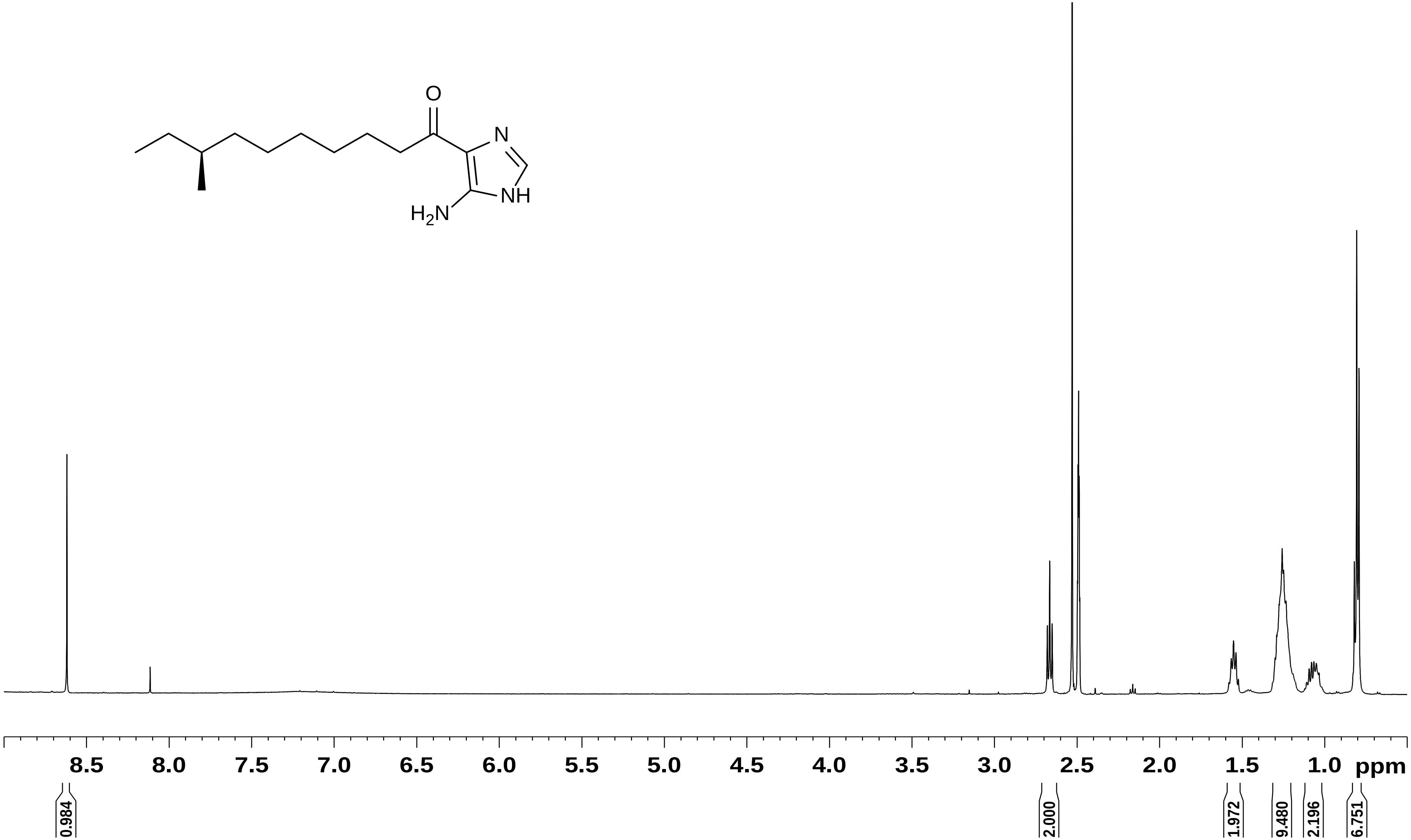

Figure S19. ^{13}C NMR spectrum of **4** (125 MHz, DMSO-d_6 with TFA)

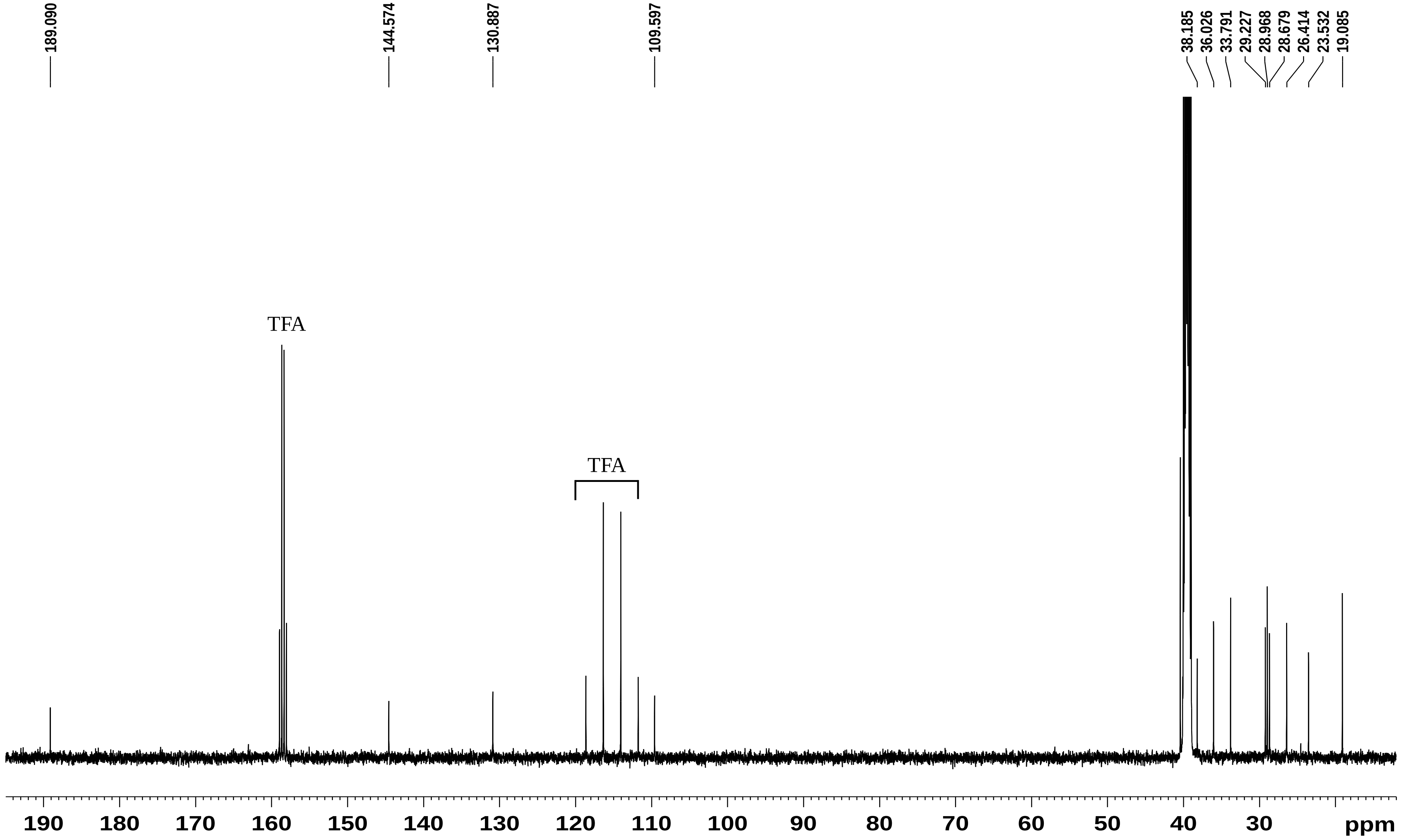

Figure S20. ^{1}H NMR spectrum of **5** (500 MHz, DMSO-d_6 with TFA)

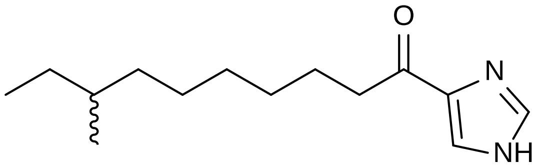

R:*S* = 1.4:98.6

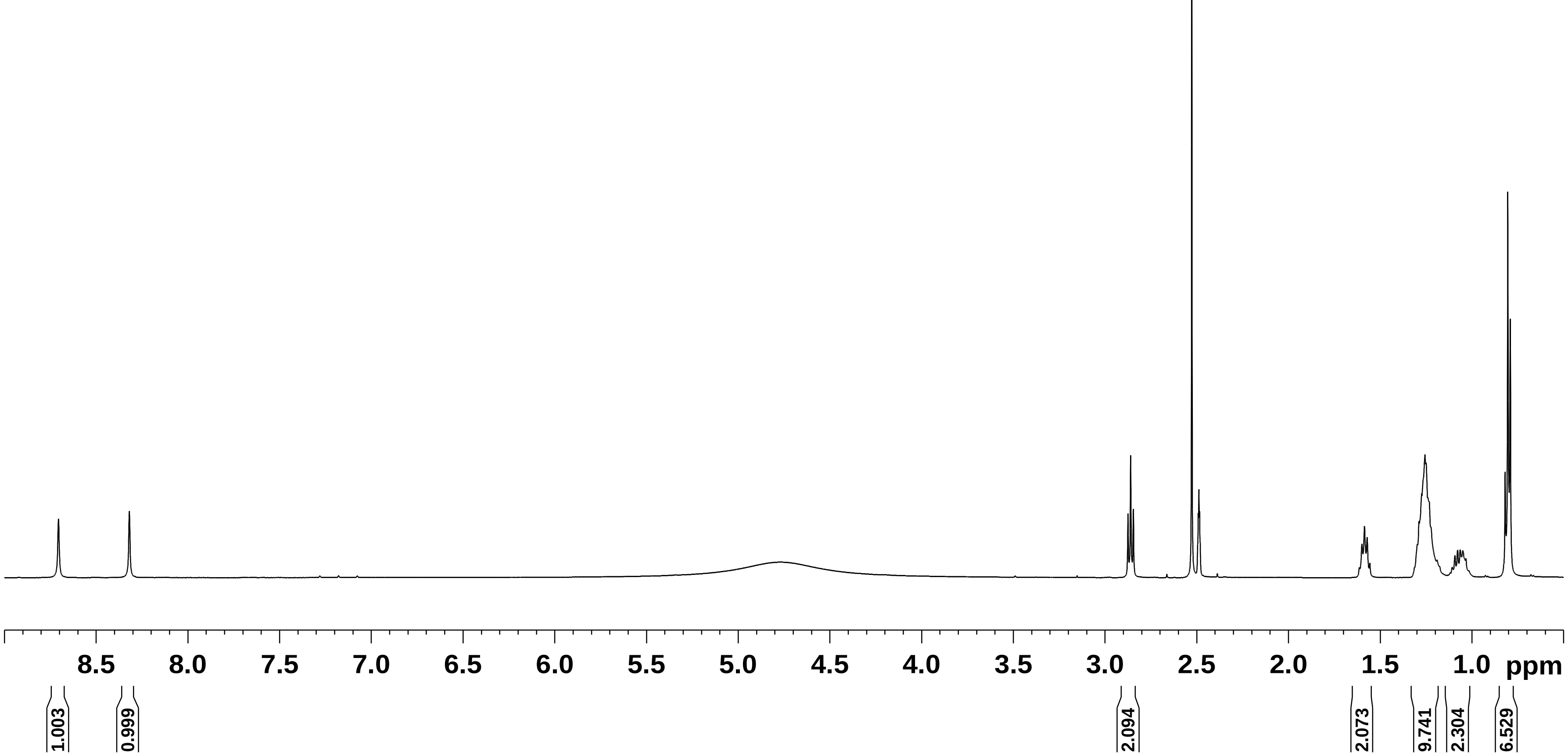

Figure S21. ^{13}C NMR spectrum of **5** (125 MHz, DMSO-d_6 with TFA)

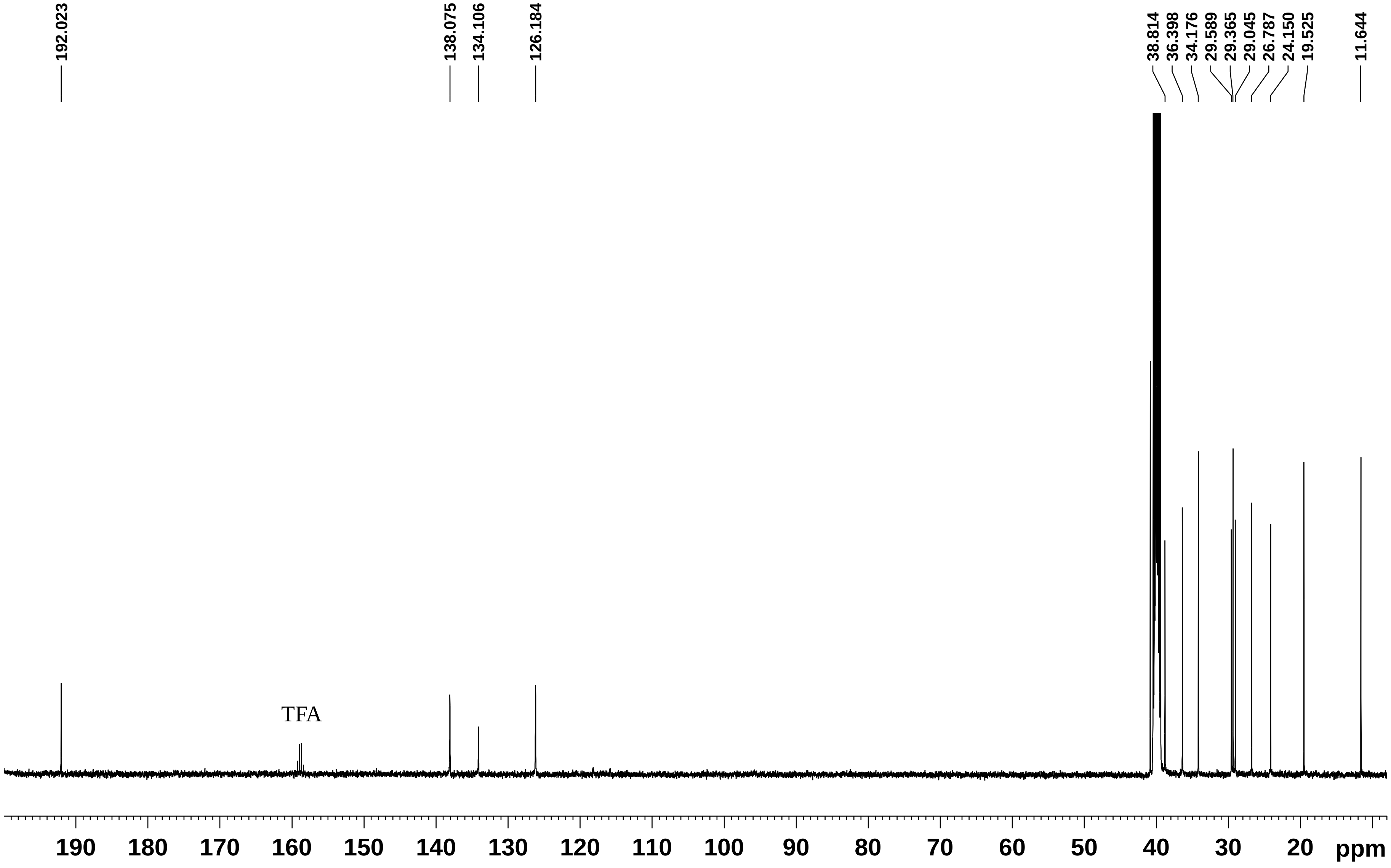

Figure S22. $^{1}J_{CH}$ HSQC spectrum of **5** (500 MHz, DMSO-d_6 with TFA)

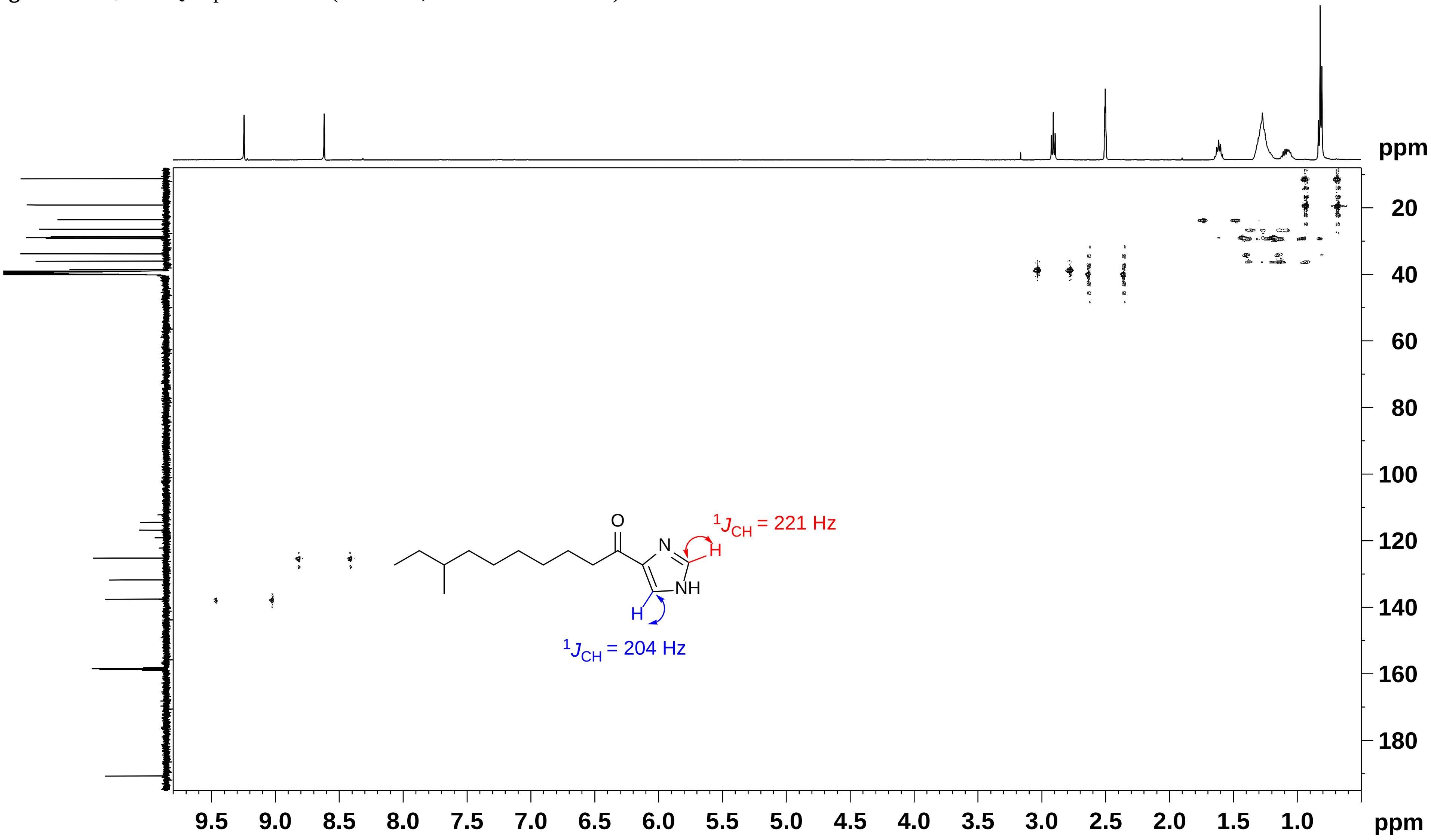

Figure S23. ^{1}H NMR spectrum of **8** (500 MHz, DMSO-d_6 with TFA)

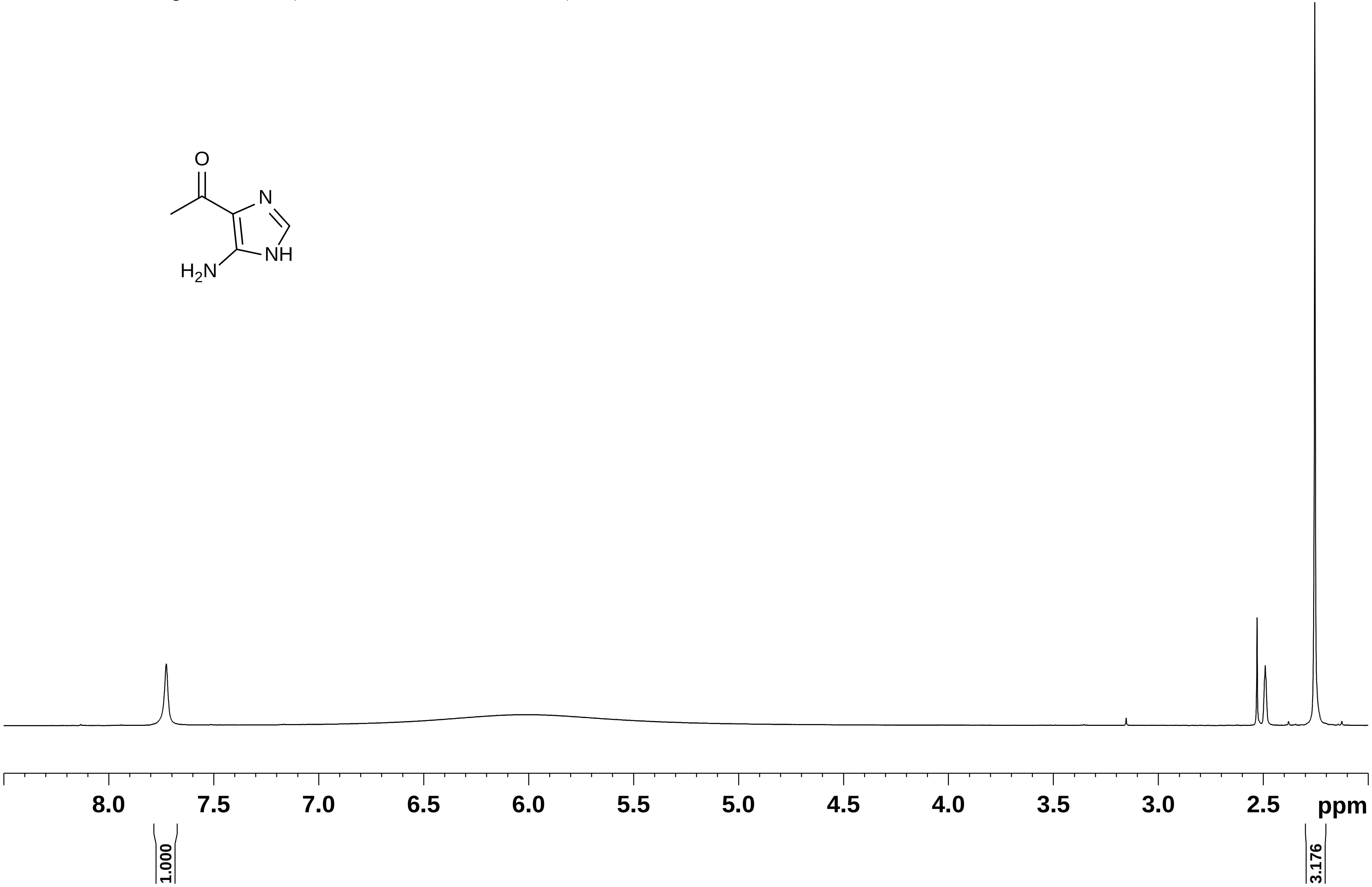

Figure S24. ^{13}C NMR spectrum of **8** (125 MHz, DMSO-d_6 with TFA)

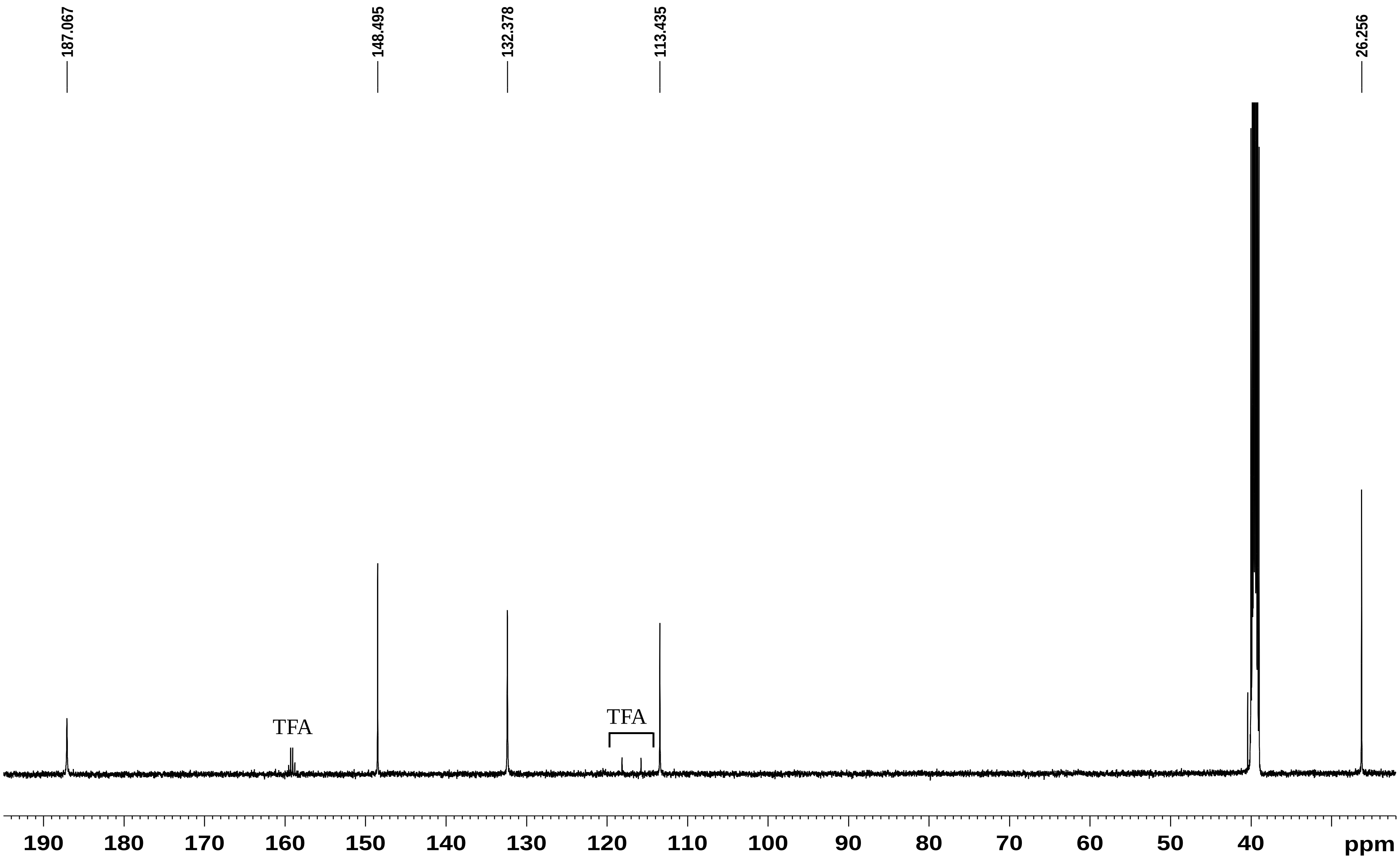

Figure S25. HSQC spectrum of **8** (500 MHz, DMSO-d_6 with TFA)

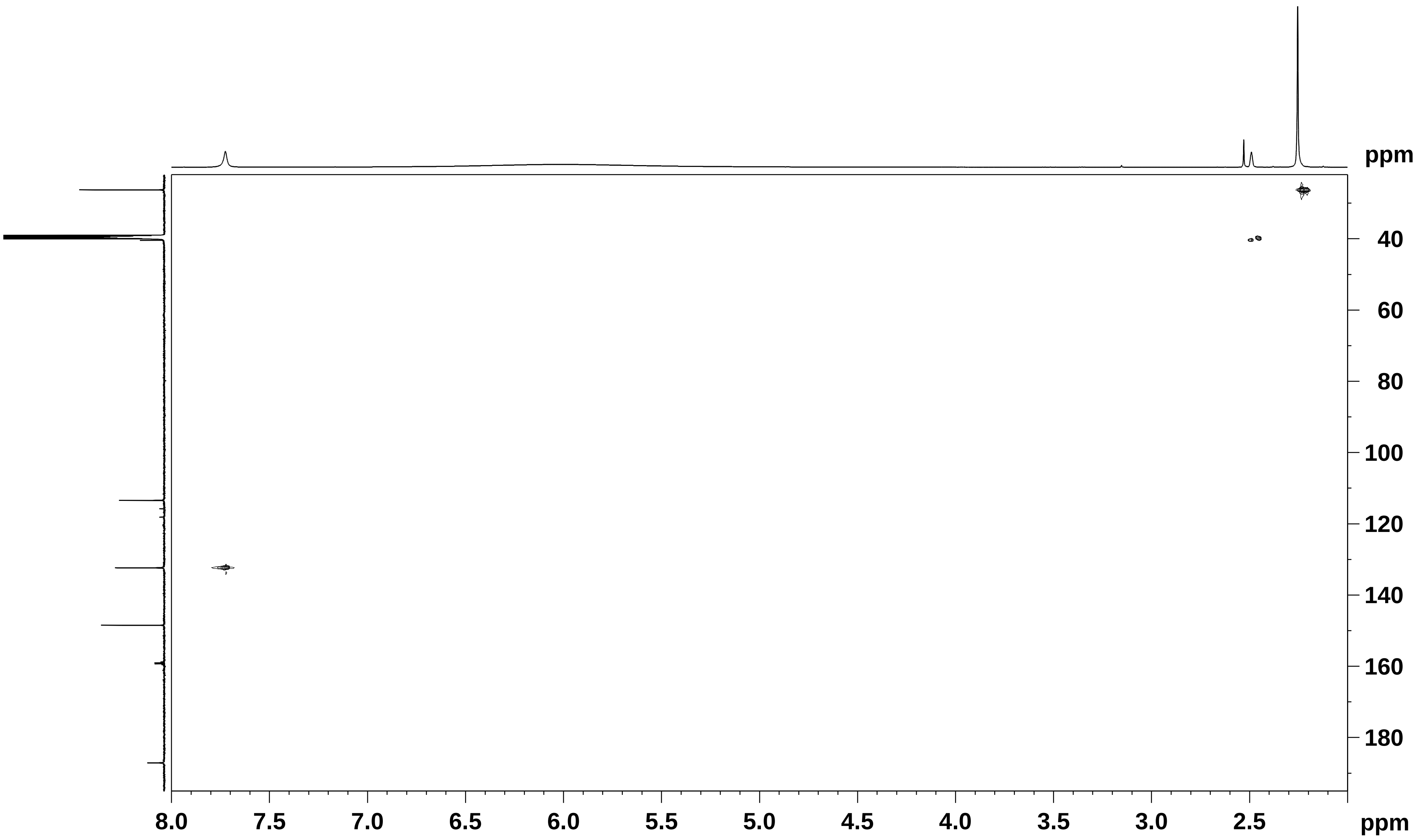

Figure S26. Coupled HSQC spectrum of **8** (500 MHz, DMSO-d_6 with TFA)

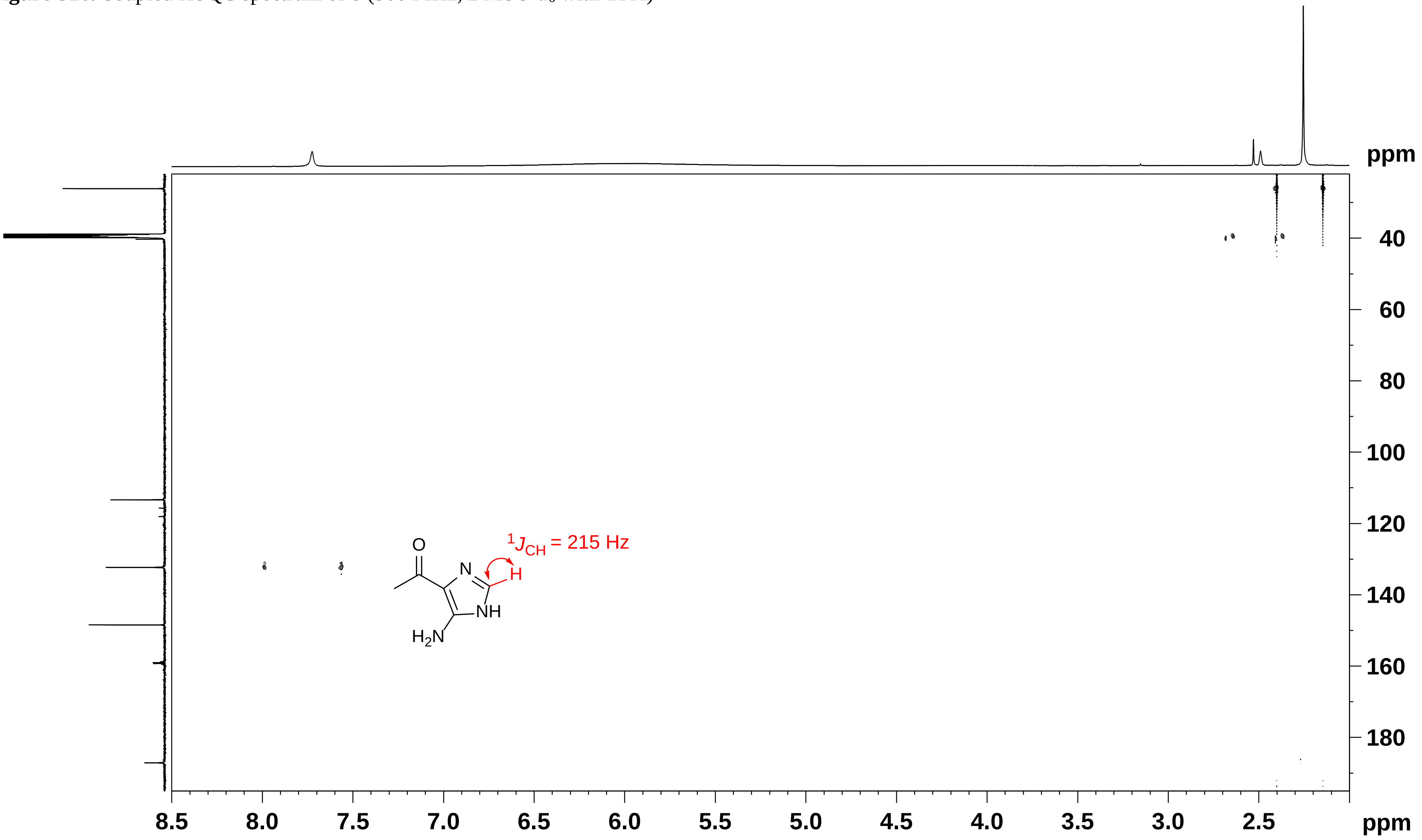

Figure S27. HMBC spectrum of **8** (500 MHz, DMSO-d_6 with TFA)

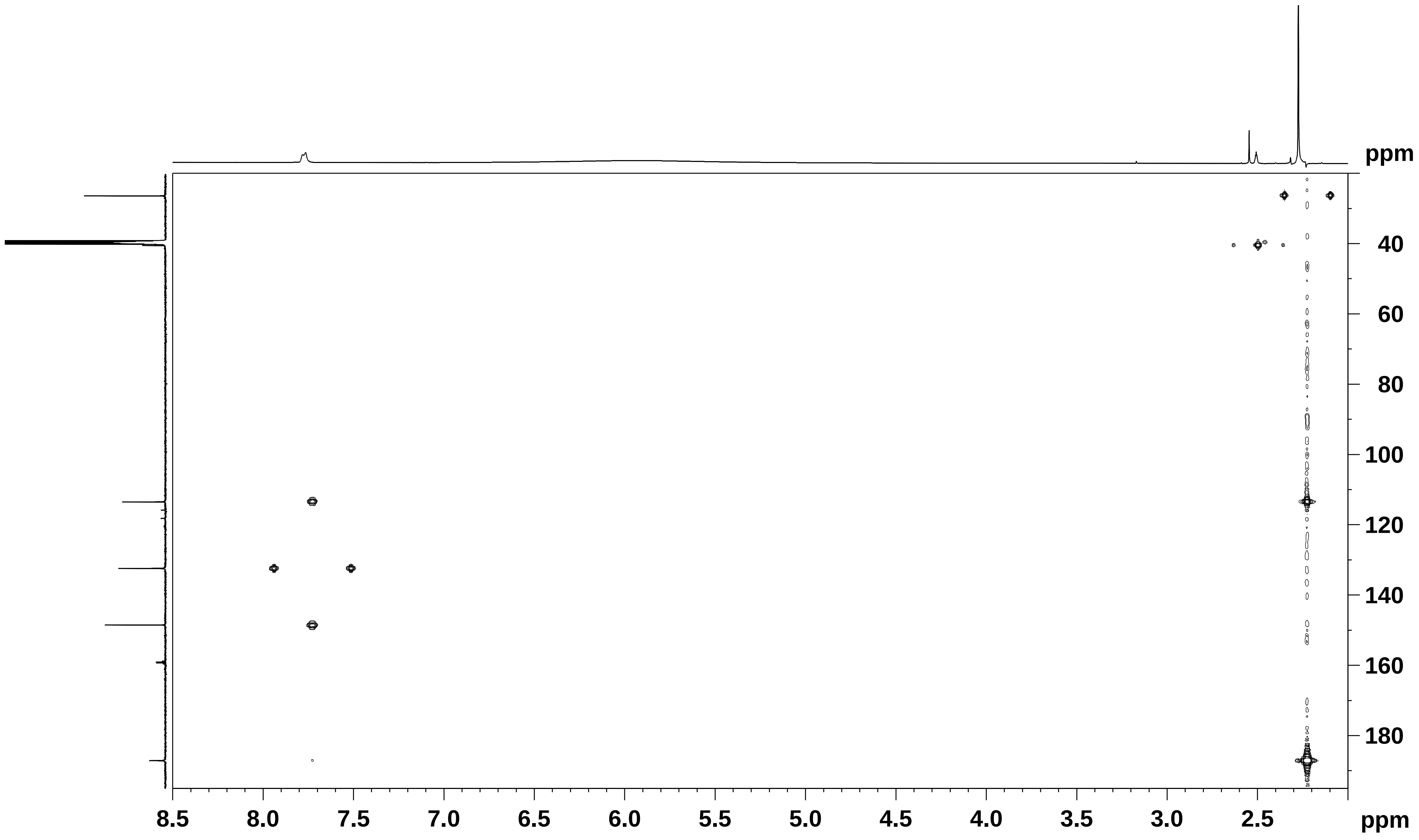

Figure S28. ^{1}H NMR spectrum of **9** (500 MHz, DMSO-d_6 with TFA)

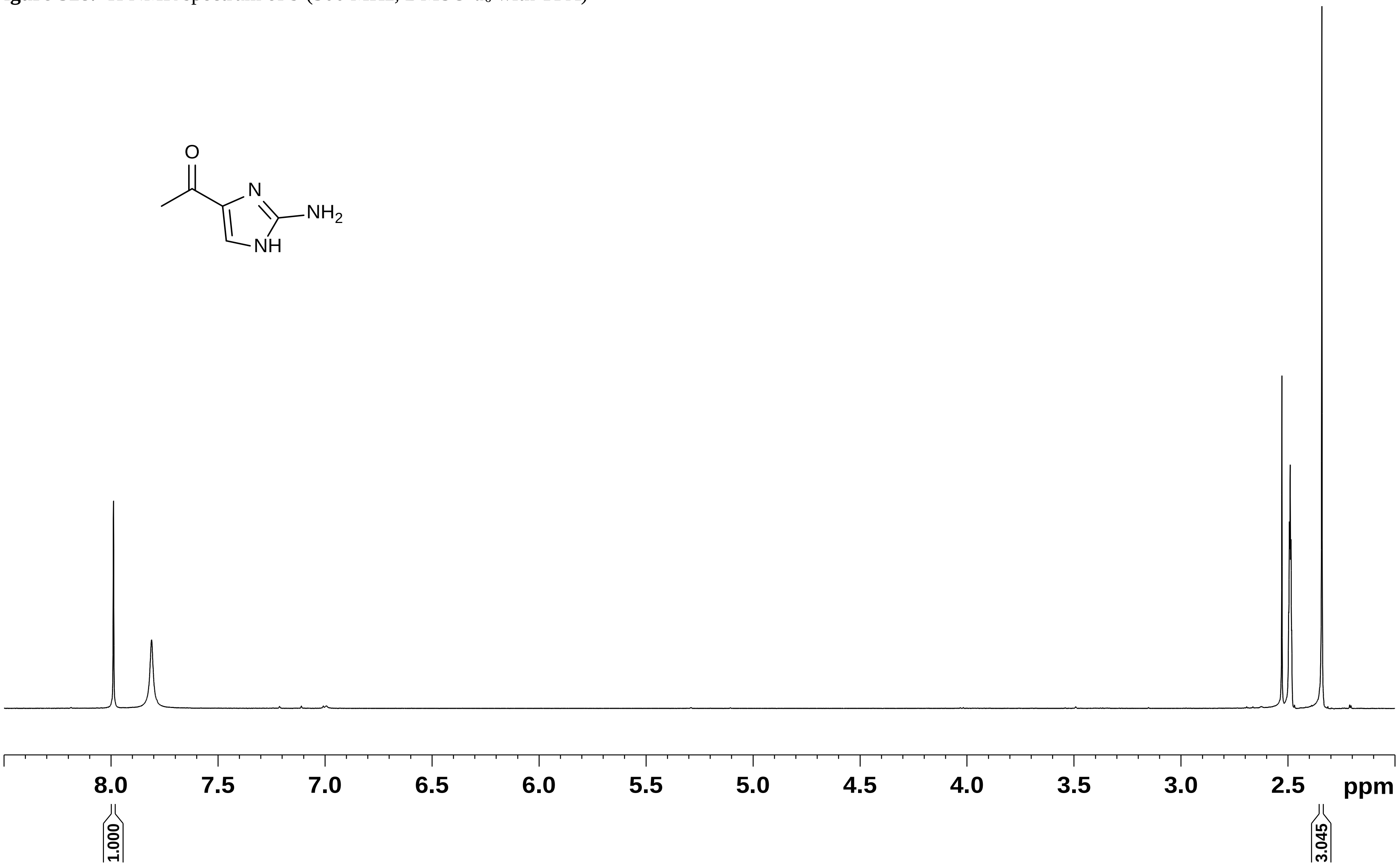

Figure S29. ^{13}C NMR spectrum of **9** (125 MHz, DMSO-d_6 with TFA)

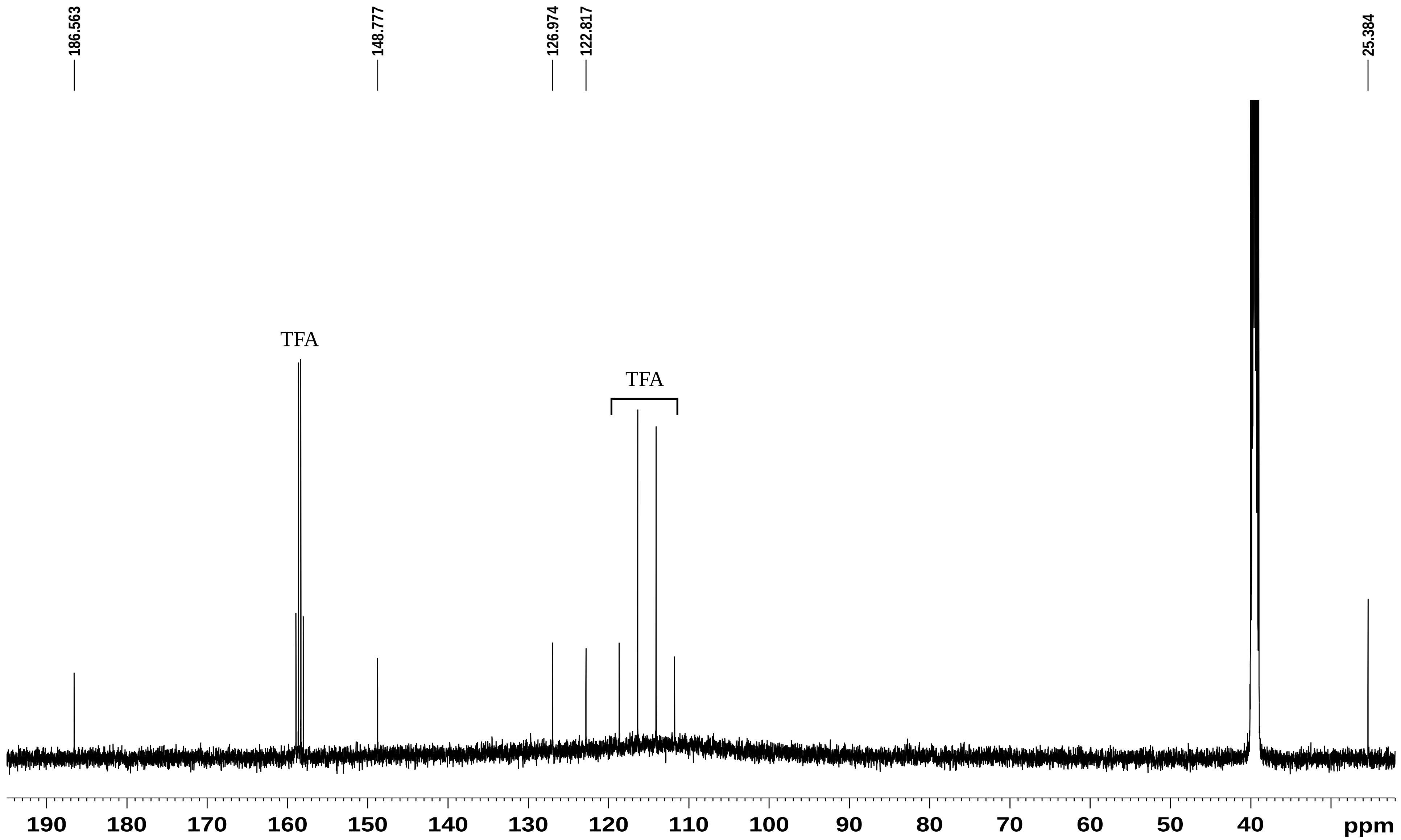

Figure S30. HSQC spectrum of **9** (500 MHz, DMSO-d_6 with TFA)

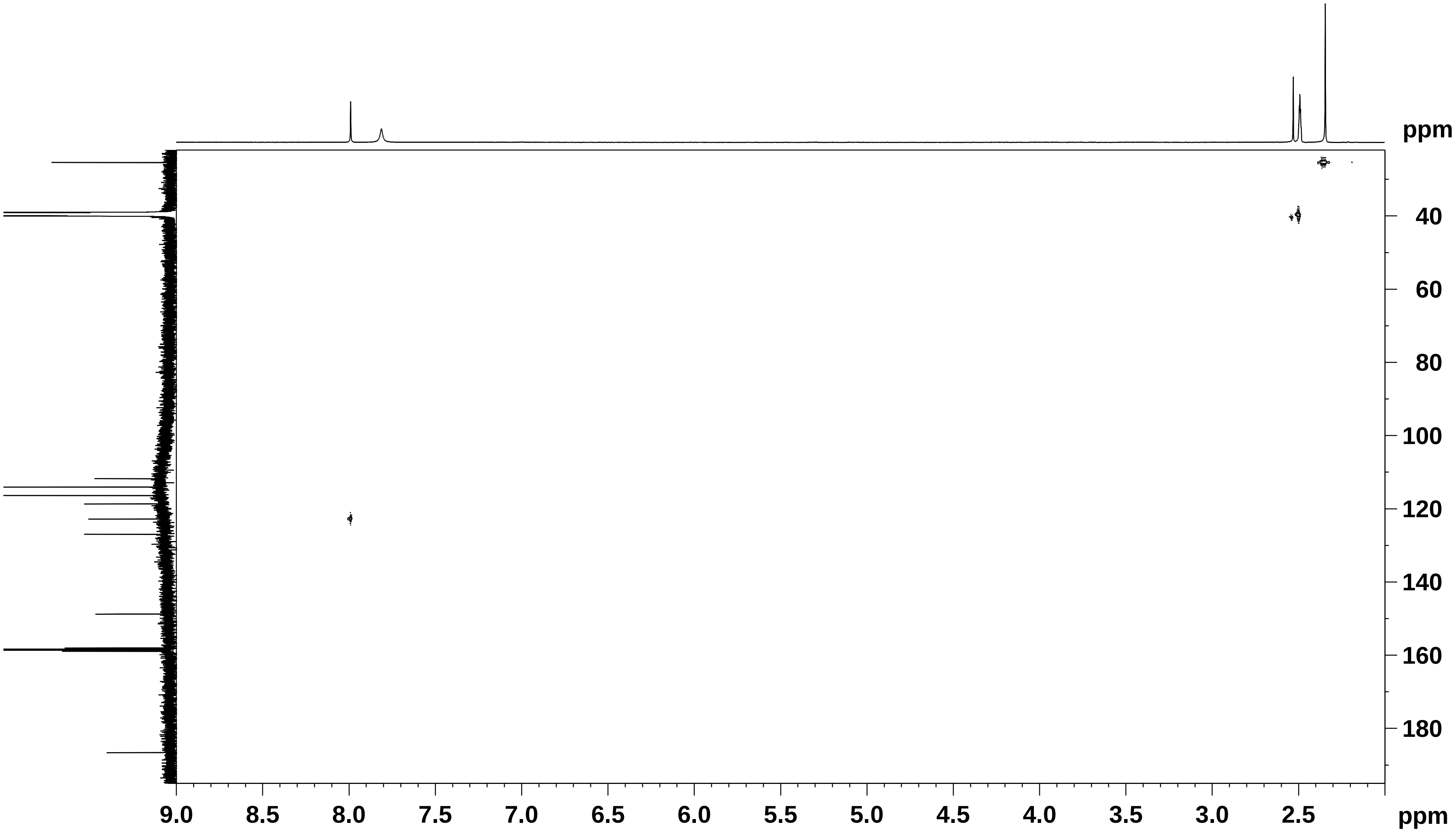

Figure S31. Coupled HSQC spectrum of **9** (500 MHz, DMSO-d_6 with TFA)

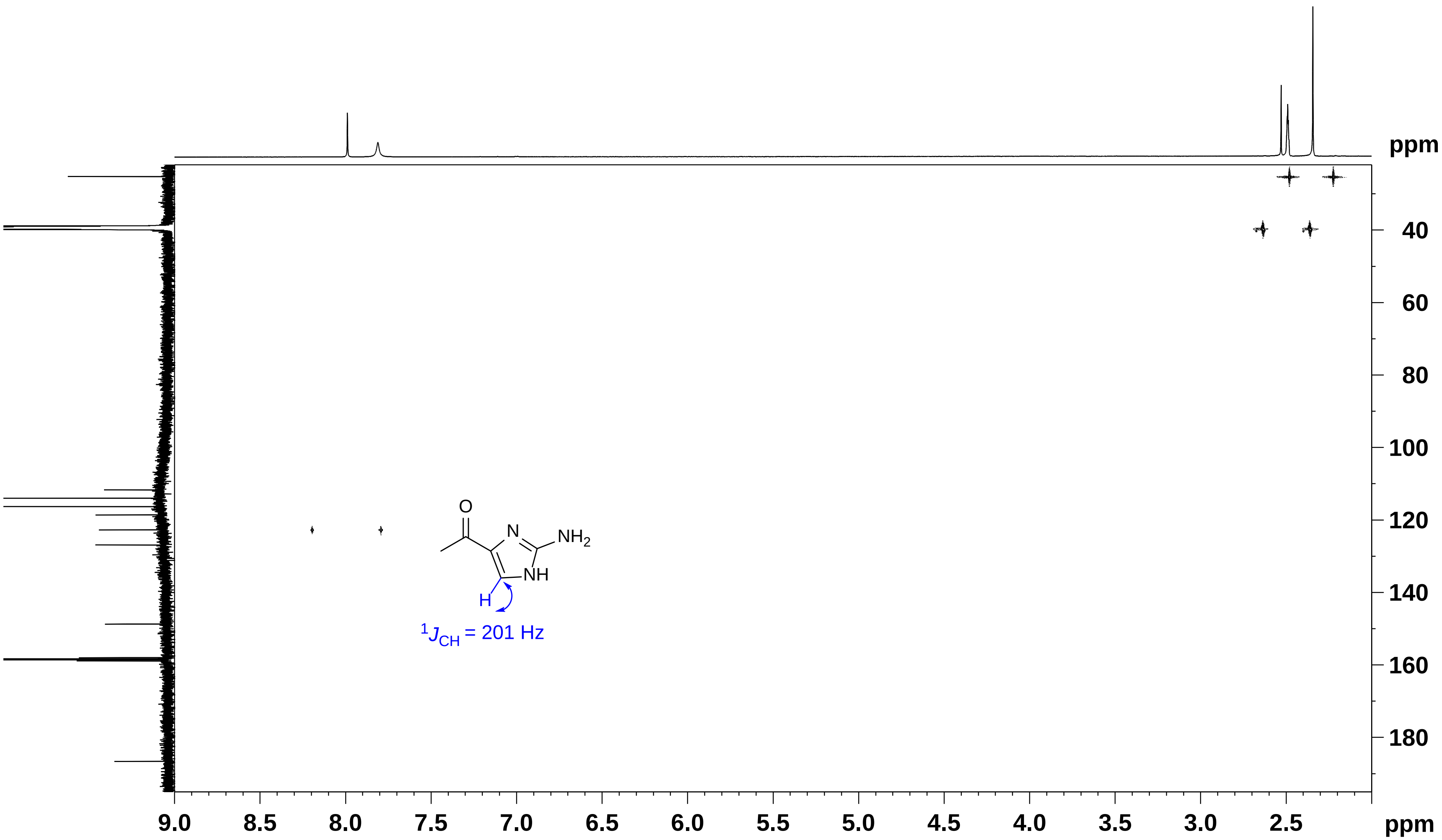

Figure S32. HMBC spectrum of **9** (500 MHz, DMSO-d_6 with TFA)

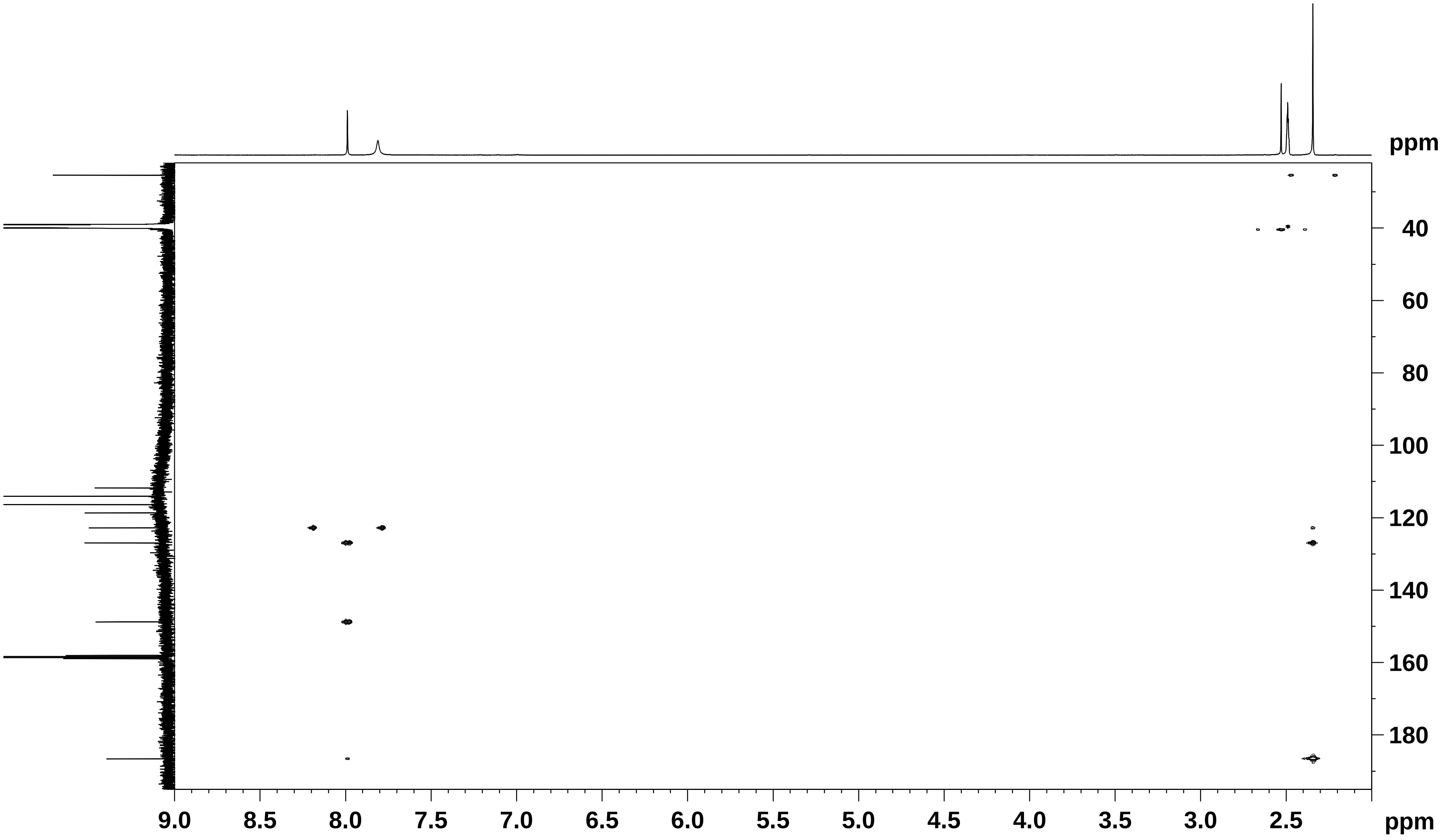

Figure S33. ^{1}H NMR spectrum of *nat*-**10**-(*R*)-2A1P (500 MHz, $CDCl_3$)

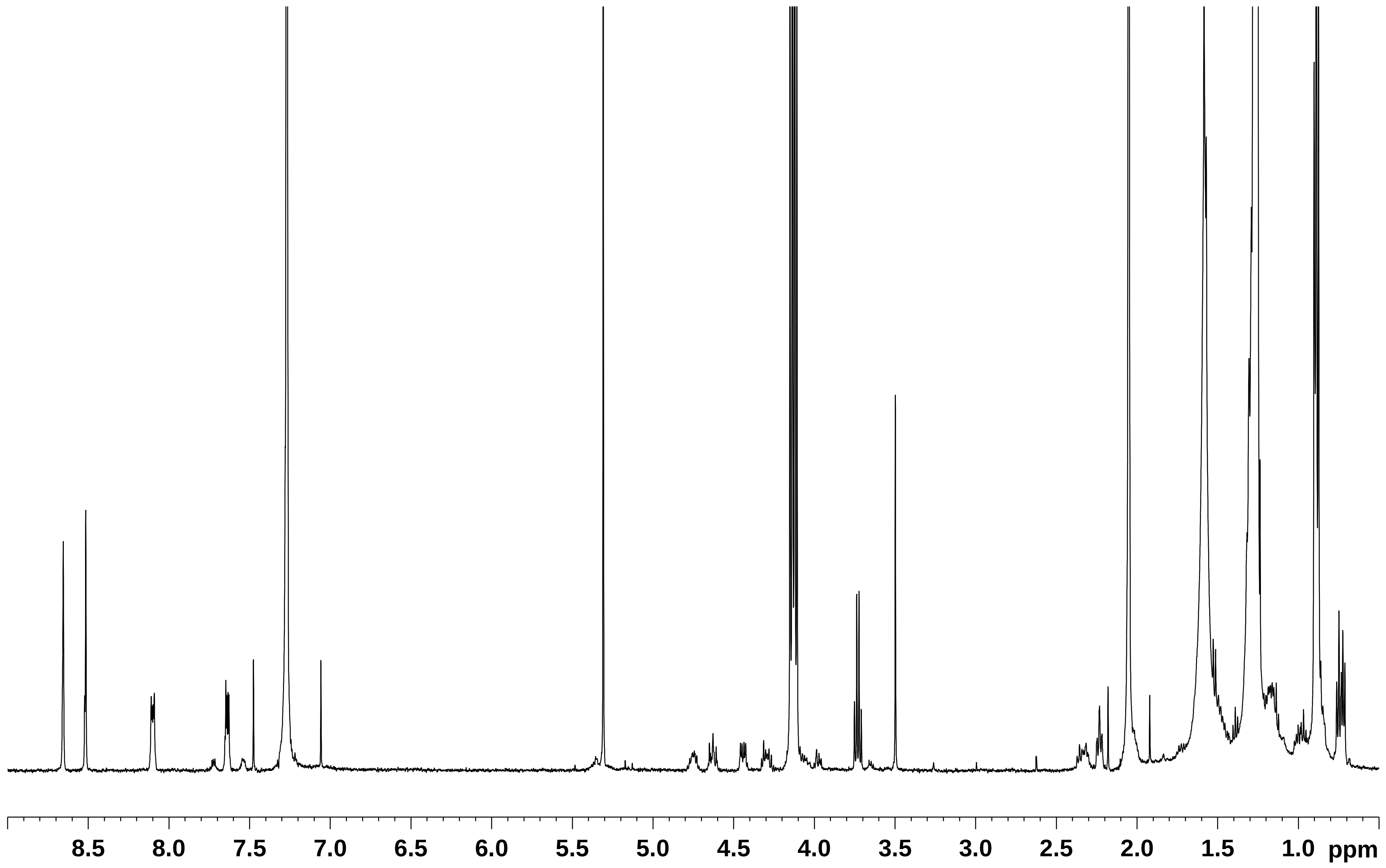

Figure S34. ^{1}H NMR spectrum of authentic (*S*)-**10**-(*R*)-2A1P (500 MHz, $CDCl_3$)

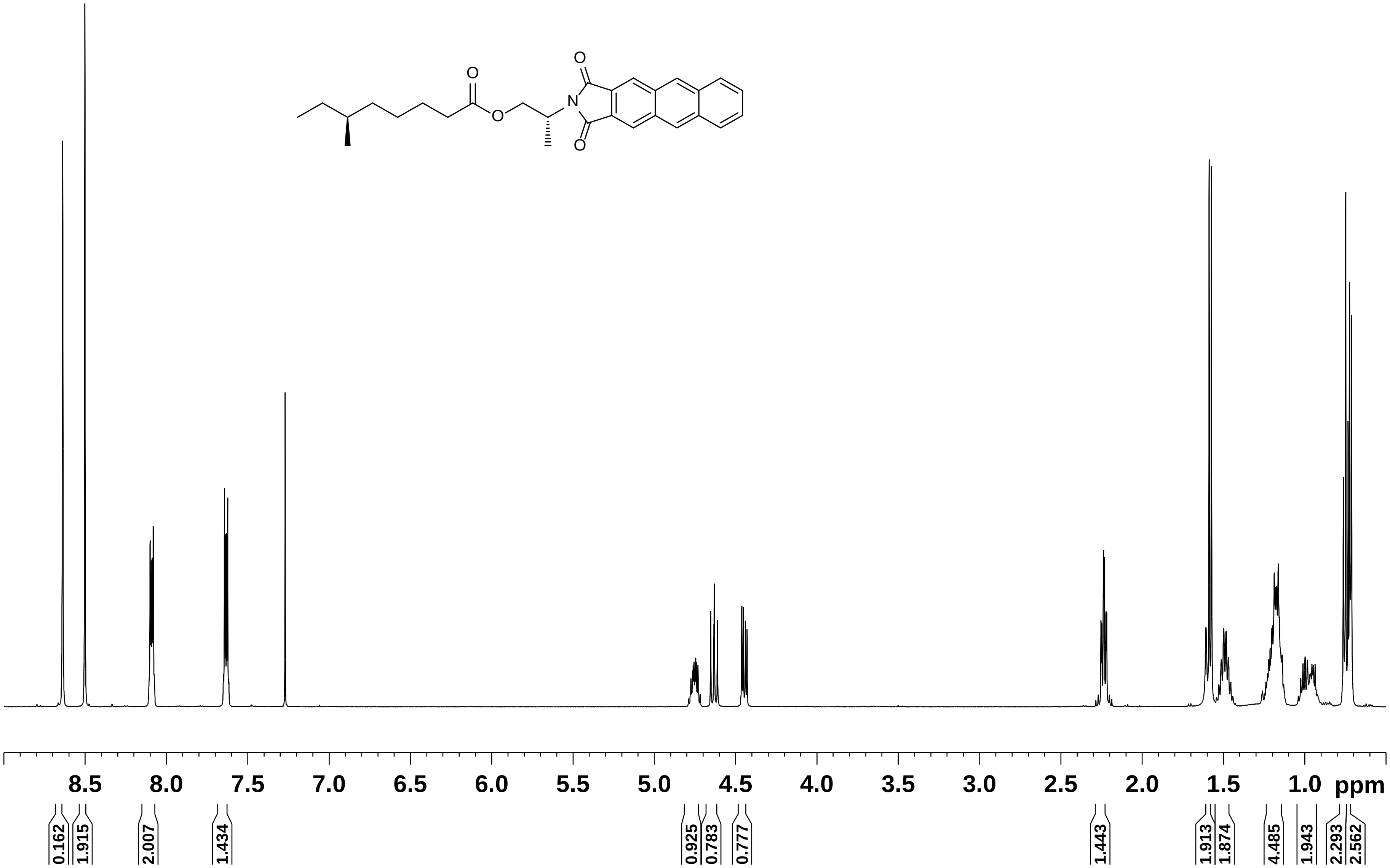

Figure S35. ^{1}H NMR spectrum of authentic (*S*)-**10**-(*S*)-2A1P (500 MHz, $CDCl_3$)

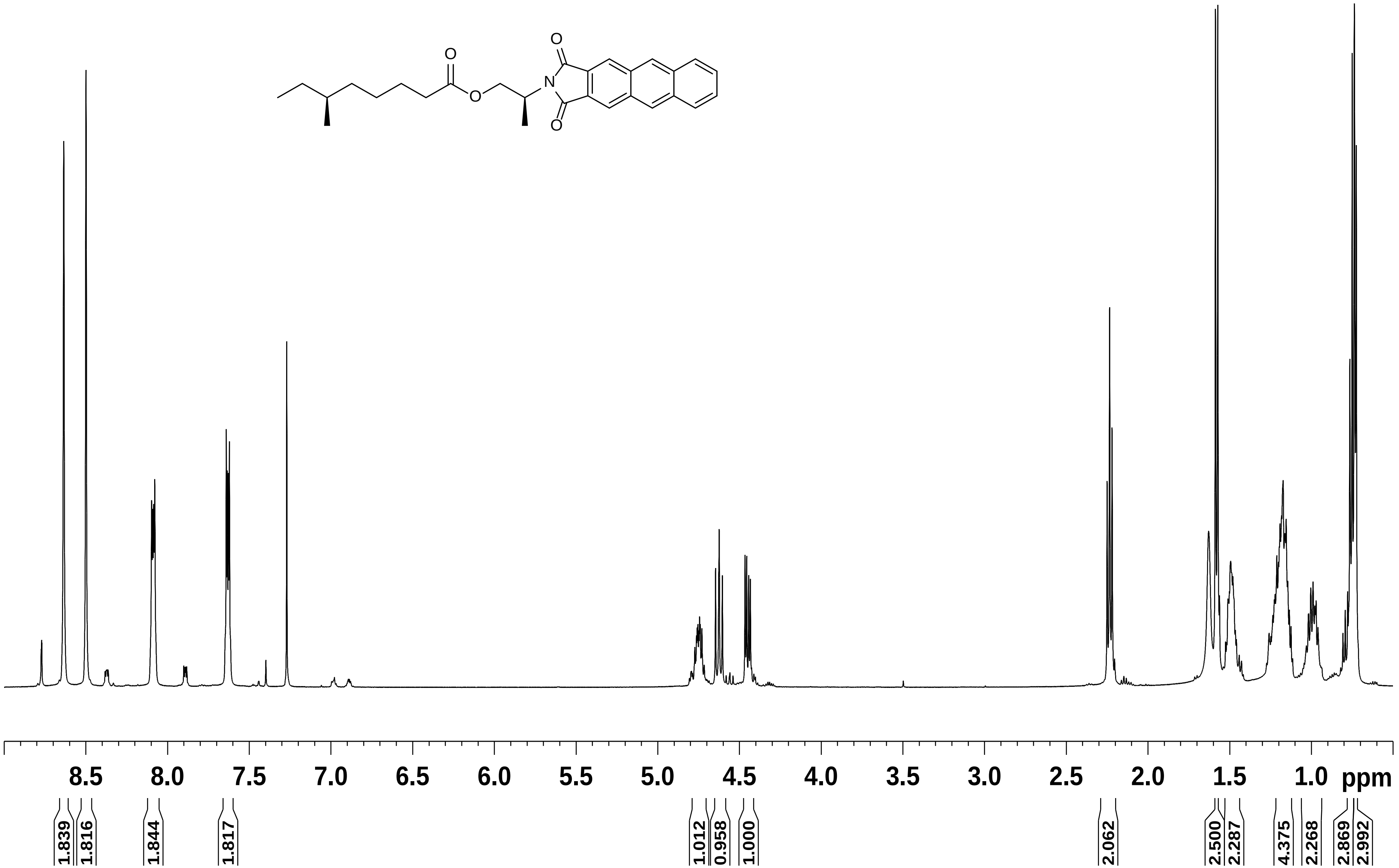

Figure S36. ^{1}H NMR spectrum of *nat*-**11**-(*R*)-2A1P (500 MHz, $CDCl_3$)

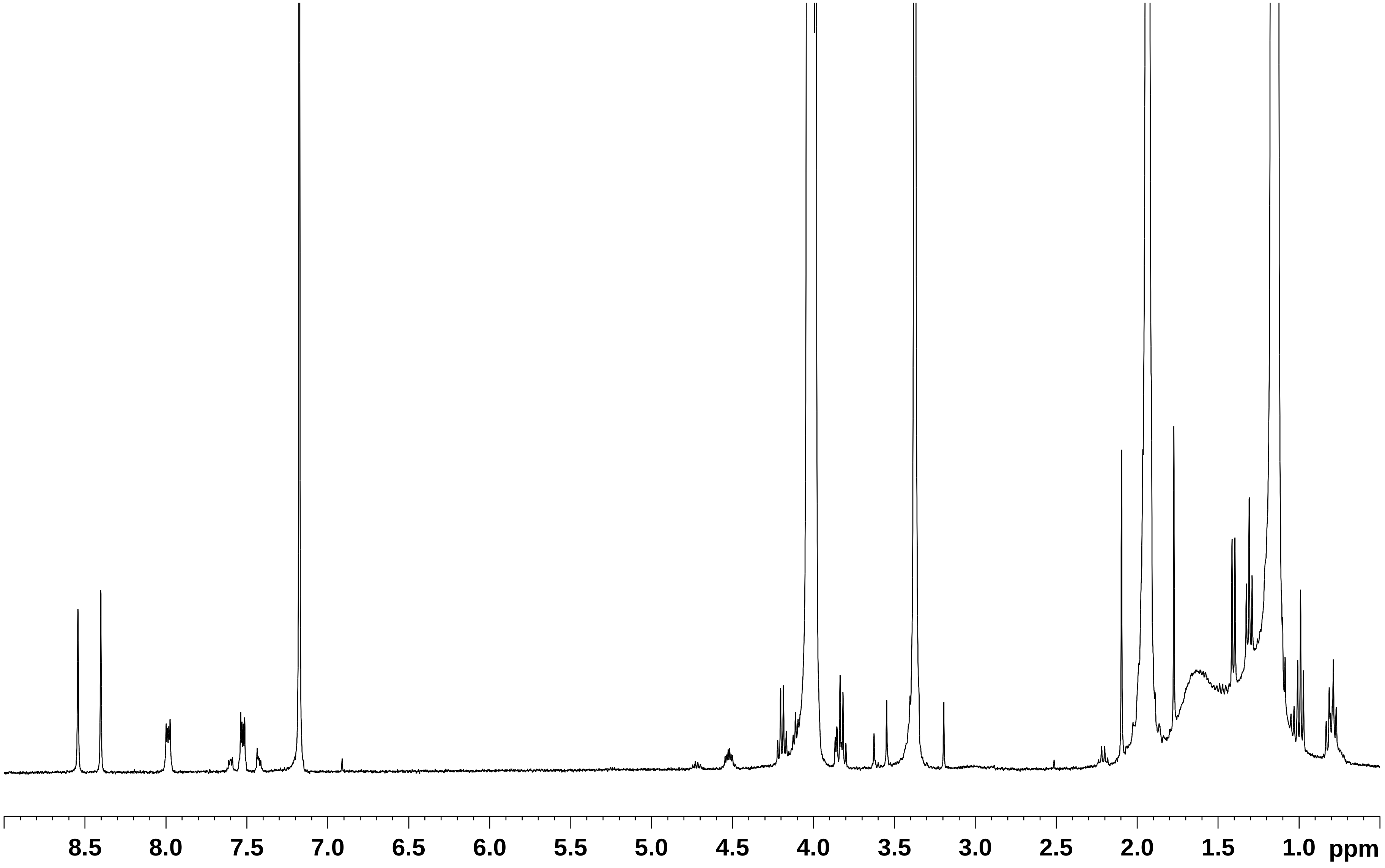

Figure S37. ^{1}H NMR spectrum of *nat*-**12**-(*R*)-2A1P (500 MHz, $CDCl_3$)

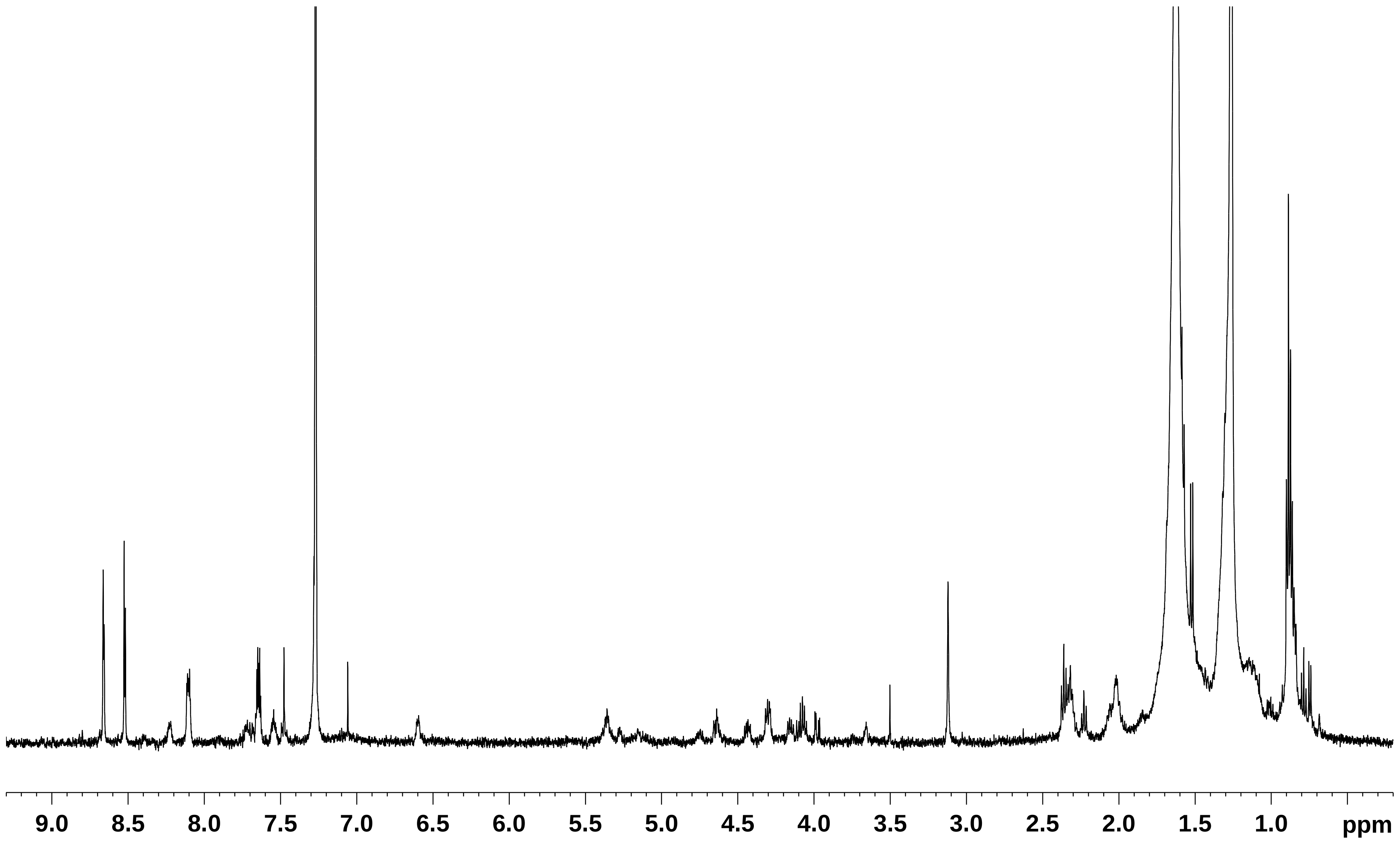

Figure S38. ^{1}H NMR spectrum of authentic (*S*)-**11**-(*R*)-2A1P (500 MHz, $CDCl_3$)

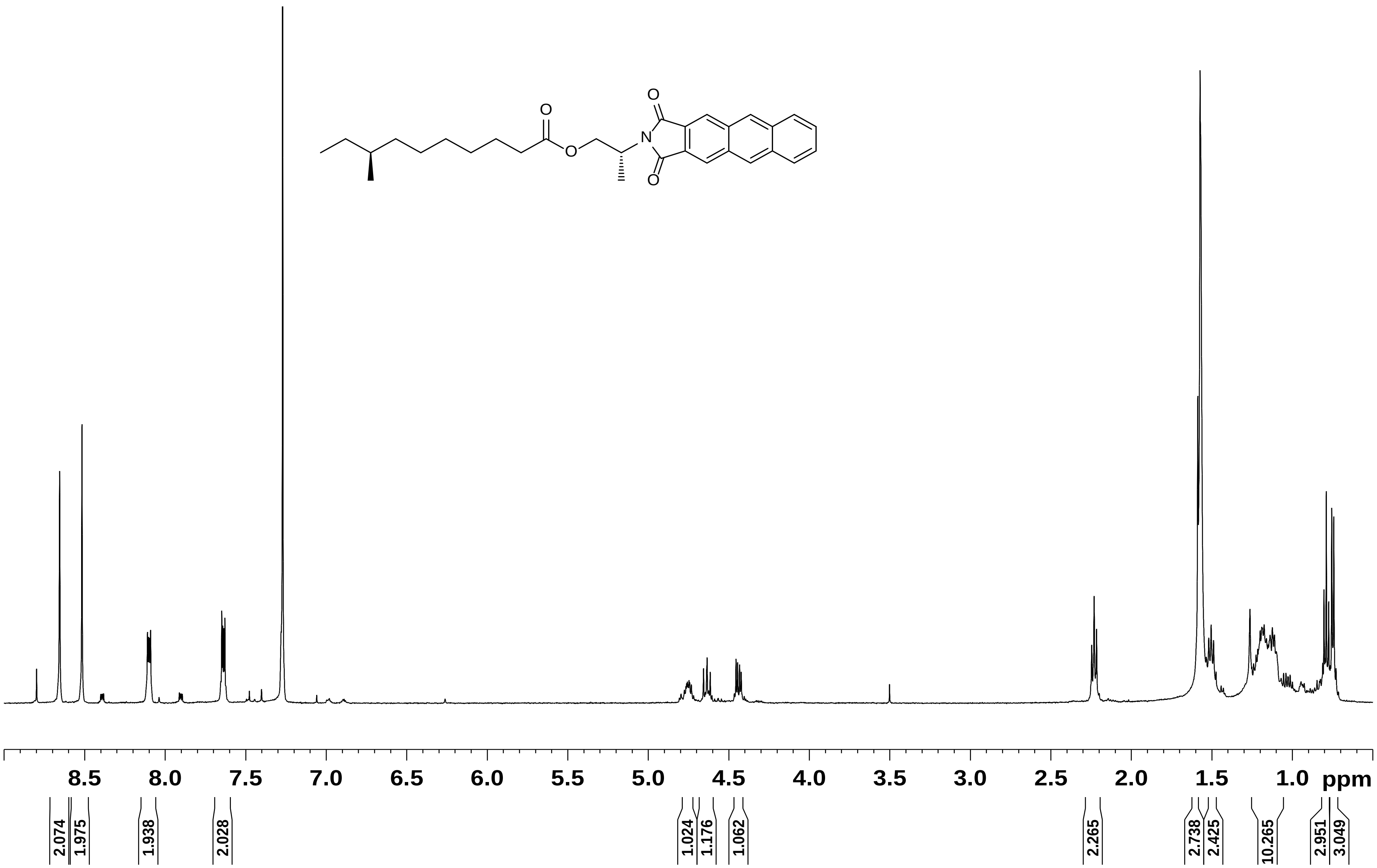

Figure S39. ^{1}H NMR spectrum of authentic (*S*)-**11**-(*S*)-2A1P (500 MHz, $CDCl_3$)

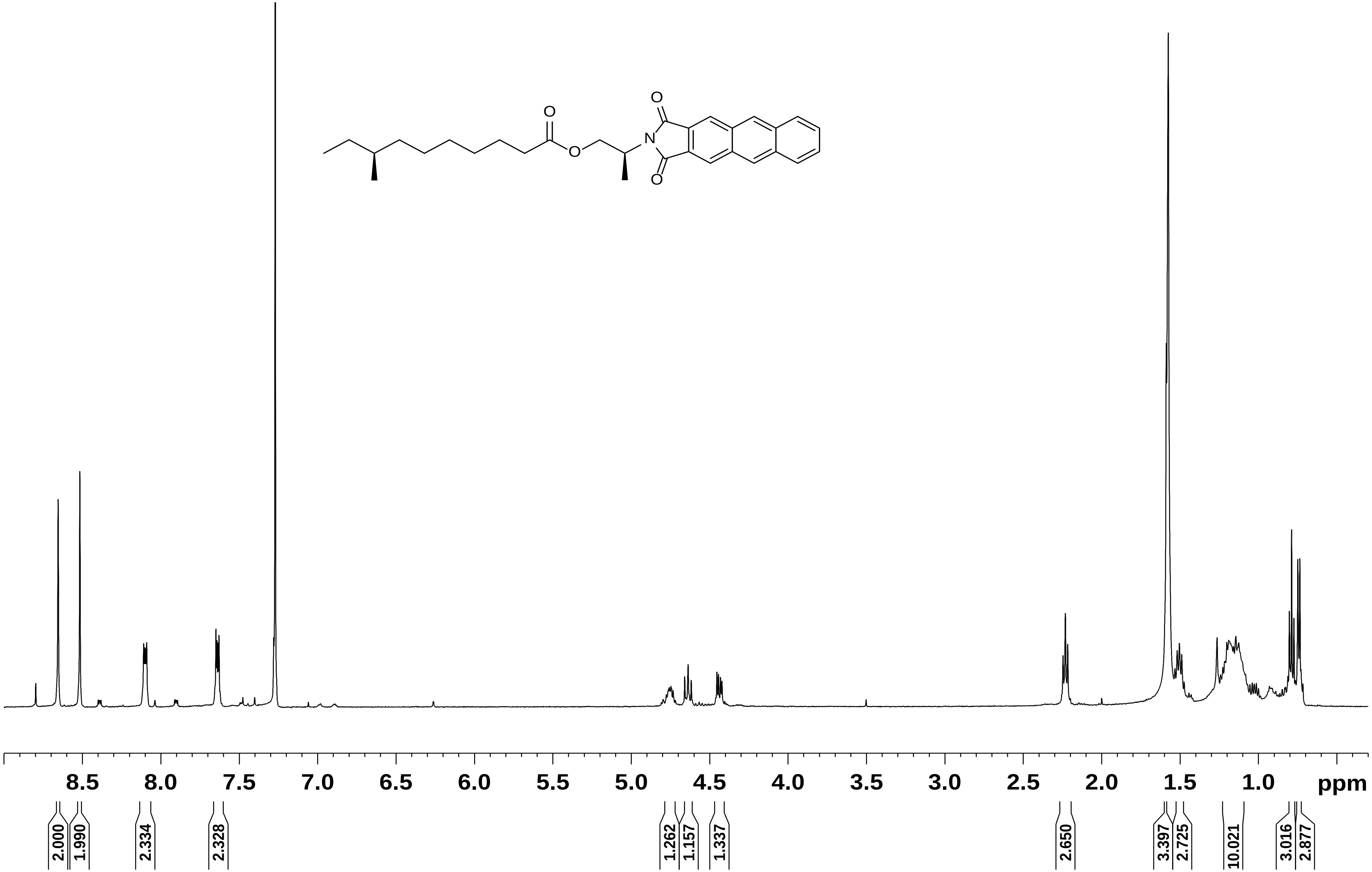

Supplement: File 1 — Copies of the NMR spectra for compounds 1 and 2. [file Beilstein_J_Org_Chem-16-2719-s001.pdf]
